# Precision fragment addition: domain-specific DeepFrag2 models for smarter lead optimization

**DOI:** 10.1039/d5dd00425j

**Published:** 2026-02-25

**Authors:** César R. García-Jacas, Harrison Green, Shayne D. Wierbowski, Jacob D. Durrant

**Affiliations:** a Department of Biological Sciences, University of Pittsburgh Pittsburgh PA USA durrantj@pitt.edu; b Machine Learning and Computational Sciences, Pfizer Research and Development Cambridge MA USA

## Abstract

This study introduces a series of machine-learning models based on DeepFrag, our previously published tool designed to guide small-molecule lead optimization through fragment addition. We demonstrate enhanced accuracy by training new DeepFrag models to predict optimizing fragments with specific sizes and chemical properties. Additionally, we show that DeepFrag accuracy improves when fine-tuned on specific receptor classes. These targeted models should prove valuable for medicinal chemists with predetermined insights into suitable molecular fragment characteristics (such as preferred size ranges, charge states, or aromaticity) or those conducting optimization campaigns against specific drug-target classes with many known ligands. To encourage adoption, we release DeepFrag2 under the open-source MIT license. Interested users can download DeepFrag2 free of charge without registration from https://durrantlab.com/deepfrag2/.

## Abstract 2

DeepFrag2, a machine-learning tool for lead optimization *via* fragment addition, is more accurate when trained on fragments with specific sizes, charge states, or aromaticity. Fine-tuning on specific receptor classes further boosts performance.

## Graphical abstract

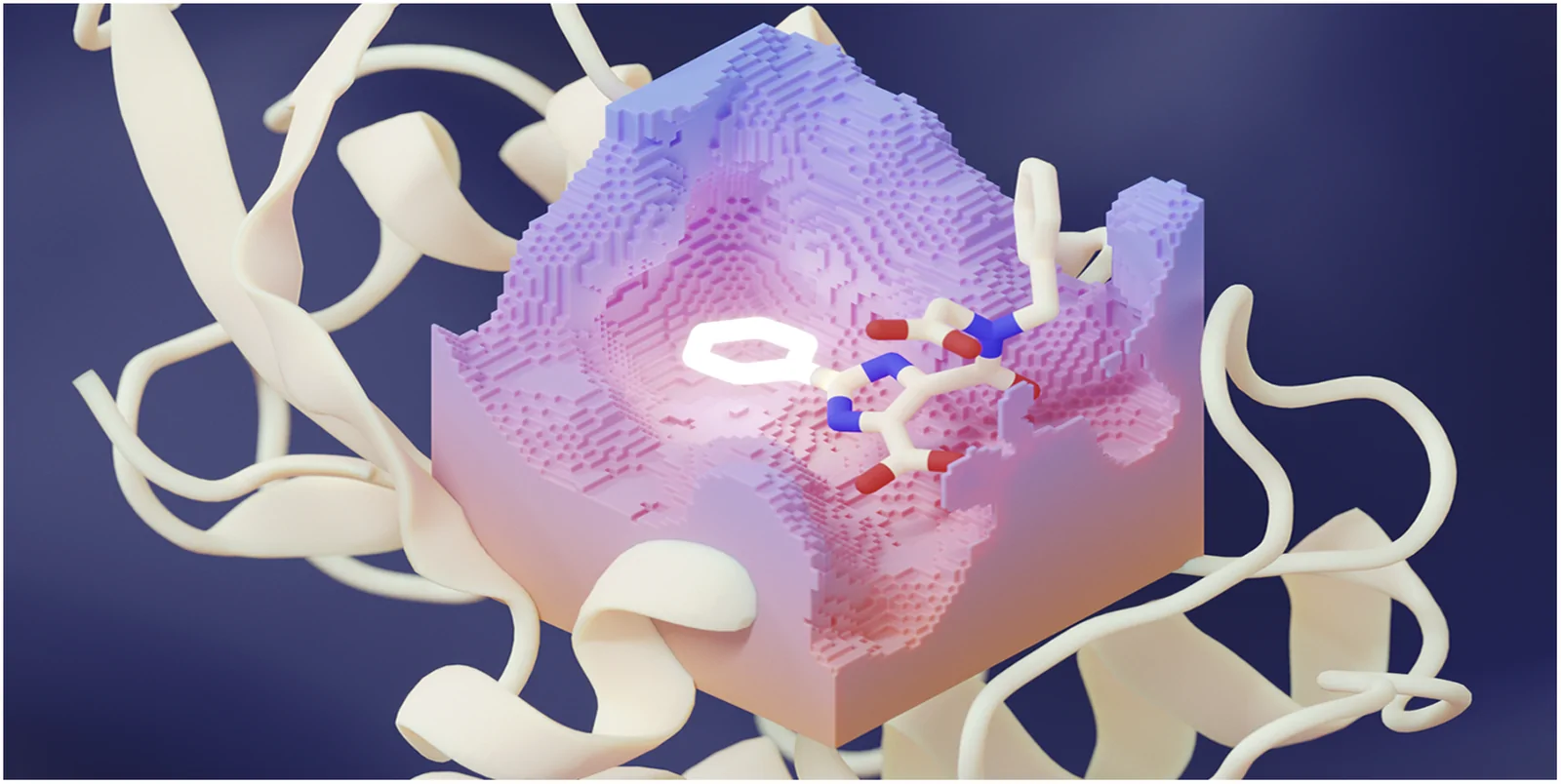

## Introduction

Drug discovery often starts by identifying bioactive molecules that bind to target proteins. Researchers find these initial “hits” through methods such as high-throughput and virtual screening. Though hit compounds serve as valuable proofs of principle, they frequently lack the properties necessary for clinically approved drugs, such as proper potency, absorption, distribution, metabolism, excretion, and low toxicity.

Lead optimization aims to improve these properties by adding, subtracting, swapping, or merging chemical moieties while maintaining target binding. Though critical, this essential process is often tedious and time-consuming, requiring medicinal chemists to synthesize and evaluate many chemical analogs. Computational tools have the potential to streamline the lead optimization process, substantially accelerating early-stage drug discovery.

DeepFrag is a machine-learning model we released in 2021 that suggests suitable lead-optimization strategies.^1,2^ As input, DeepFrag accepts (1) the structure of a small-molecule ligand bound to a protein receptor and (2) the location of a ligand atom where an optimizing fragment should be added. It then predicts a vector (topological fingerprint) describing a candidate fragment to add at that position. By comparing this predicted fingerprint to a library of fragments with known fingerprints, one can select suitable fragments that may improve binding. Although DeepFrag was trained specifically for fragment addition, researchers can also use it to perform fragment replacement by first removing an existing fragment and then predicting a suitable substitution. Indeed, fragment replacement may well be the more common application in typical lead-optimization workflows.

The original DeepFrag model is useful for optimizing ligands that bind to a wide range of protein receptors. This versatility is a product of the training approach, which leveraged the full Binding MOAD (2019 release)^3–5^ with its many diverse protein and ligand classes. However, the original DeepFrag performs better when predicting smaller fragments due to their overrepresentation in the training data and the inherently smaller chemical space of fragments with few heavy atoms. Further, the original DeepFrag does not leverage expert (human) knowledge to intelligently select fragments with specific physicochemical properties and cannot be tailored to specific drug-target classes.

To address these limitations, we developed a more flexible DeepFrag codebase that enables three complementary approaches to improve prediction accuracy. We used this new codebase to first create separate “targeted” models for fragments with (1) at most three heavy atoms and (2) at least four heavy atoms, addressing the original model's bias in favor of smaller, often less pharmacologically valuable fragments. Second, we trained targeted models on fragments with distinct chemical properties (*i.e.*, acidic/basic or aromatic/aliphatic) so expert users can select suitable models appropriate for their specific fragment requirements. Third, we leveraged the more flexible codebase to fine-tune models for specific protein classes, producing new models with improved accuracy in their respective domains.

To encourage adoption, we release our software under the open-source MIT license, available for free download without registration at https://durrantlab.com/deepfrag2/. The same webpage links to a Google Colab that demonstrates use. We hope these new models will help researchers generate lead compounds faster, decreasing the time and cost associated with lead optimization.

## Materials and methods

### Fragment-specific (targeted) DeepFrag2 models

#### Dataset preparation

**Fig. 1:**
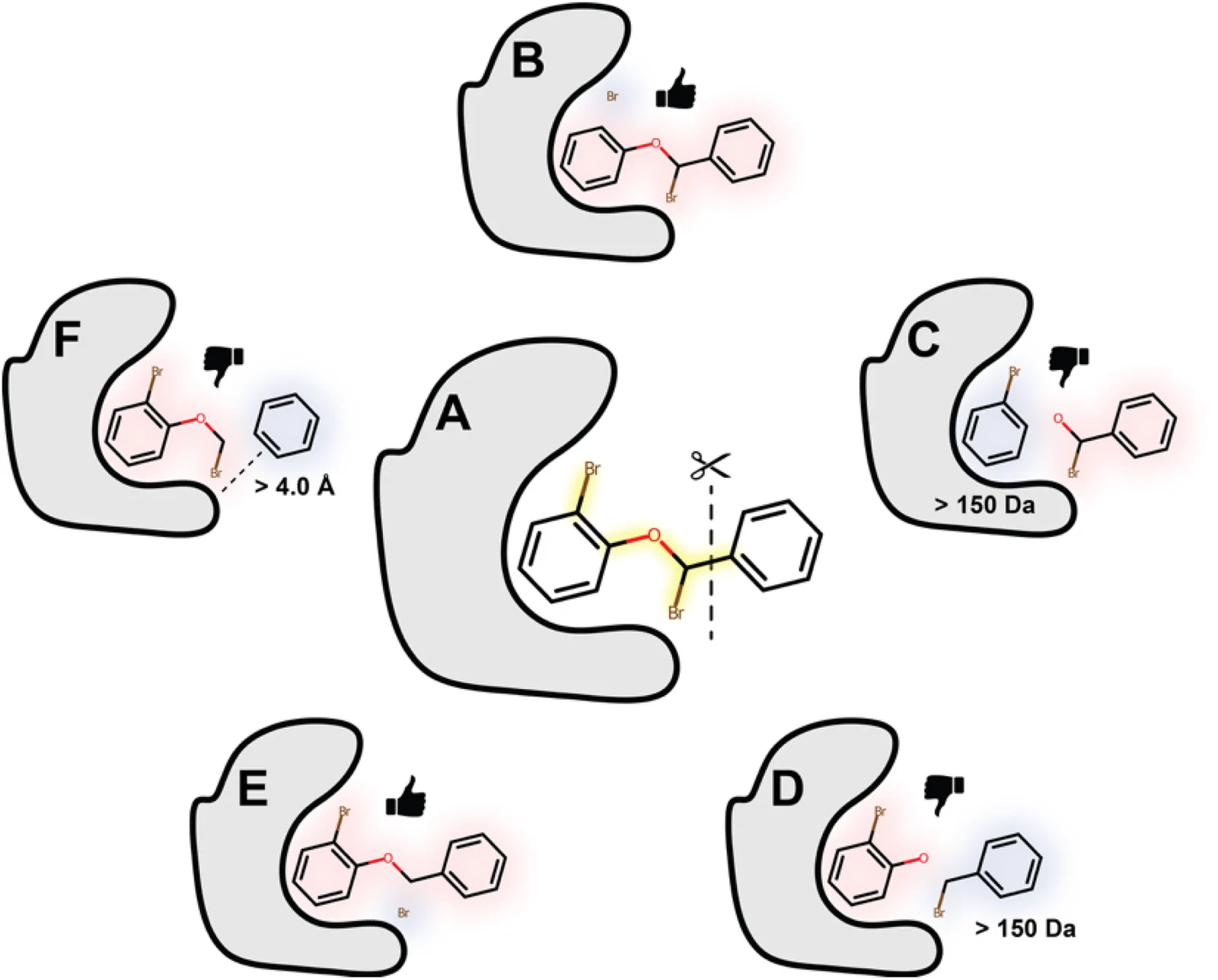
Illustration of the DeepFrag2 fragmentation scheme applied to a single protein/ligand complex. The gray blob represents the protein. (A) The five non-ring single bonds of the ligand are highlighted in yellow. These are the bonds that are separately cut, represented by scissors and a dashed line. (B–F) Each bond becomes a candidate for fragmentation, such that five potential examples result from this single protein/ligand complex. After fragmentation, we term the larger portion the “trimmed ligand” (highlighted in red). The remaining smaller portion we call the “fragment” (highlighted in blue). (C and D) Some examples are rejected (thumbs down) because the fragment has molecular weight greater than 150 Da. (F) Some examples are rejected because no fragment atom comes within 4 Å of a receptor atom. The remaining examples are retained (thumbs up).

To generate appropriate datasets for the fragment-specific (targeted) DeepFrag models (*i.e.*, models trained to predict fragments with specific physicochemical properties such as size, aromaticity, or acidity/basicity), we generally followed the same approach described in the original DeepFrag paper.^2^ For each protein/ligand complex in the Binding MOAD 2020, we identified all the ligand single bonds that did not belong to any ring (Fig. 1A, highlighted in yellow) as individual candidates for fragmentation. We separately fragmented (“cut”) along each of these single bonds. Each cut created a unique example composed of the (1) protein receptor, (2) trimmed ligand, and (3) terminal fragment. Typically, there were multiple such examples generated from each protein/ligand complex (Fig. 1B–F).

##### Initial (all) dataset

To create our initial distinct (non-overlapping) training, validation, and testing sets, we first grouped the 41 359 entries of the Binding MOAD 2020 by Binding-MOAD protein family annotation. We then randomly assigned all structures to the training, validation, or testing set using a roughly 60 : 20 : 20 split, ensuring that all structures within a given family were always assigned to the same set (Table 1, row 1).

**Table 1:** Entries (unique PDB IDs) present in the training, validation, and testing sets at different steps of dataset construction^a^

Initial (all) dataset				Relative train/val/test size (%)
Steps	Train set size (% change)	Val set size (% change)	Test set size (% change)	
(1) Original split based on protein family	24 221	9557	7581	58.6%/23.1%/18.3%
(2) Remove val/test entries with missing SMILES	24 221 (−0.0%)	9452 (−1.1%)	7466 (−1.5%)	58.9%/23.0%/18.1%
(3) Remove val/test entries with ligands also in train	24 221 (−0.0%)	5113 (−45.9%)	3332 (−55.4%)	74.1%/15.7%/10.2%
(4) Remove test entries with ligands also in val	24 221 (−0.0%)	5113 (−0.0%)	3081 (−7.5%)	74.7%/15.8%/9.5%
(5) Remove fragments with MW > 150 Da	22 722 (−6.2%)	4856 (−5.0%)	2890 (−6.2%)	74.6%/15.9%/9.5%
(6) Remove fragments with receptor distance > 4.0 Å	22 707 (−0.1%)	4854 (−0.0%)	2890 (−0.0%)	74.6%/15.9%/9.5%
(7a) Small-fragment dataset (derived from initial)	22 393 (−1.4%)	4739 (−2.4%)	2807 (−2.9%)	74.8%/15.8%/9.4%
(7b) Large-fragment dataset (derived from initial)	19 824 (−12.7%)	4349 (−10.4%)	2591 (−10.3%)	74.1%/16.2%/9.7%
(8a) Aromatic-fragment dataset (from large-fragment)	12 367 (−37.6%)	2987 (−31.3%)	1738 (−32.9%)	72.4%/17.5%/10.2%
(8b) Aliphatic-fragment dataset (from large-fragment)	16 276 (−17.9%)	3155 (−27.5%)	1925 (−25.7%)	76.2%/14.8%/9.0%
(8c) Acidic-fragment dataset (from large-fragment)	10 638 (−46.3%)	1340 (−69.2%)	789 (−69.5%)	83.3%/10.5%/6.2%
(8d) Basic-fragment dataset (from large-fragment)	3021 (−84.8%)	961 (−77.9%)	480 (−81.5%)	67.7%/21.5%/10.8%

a The initial (all) dataset includes entries with both large and small fragments. Entries with proteins belonging to the same Binding-MOAD protein family are always present in the same split. Similarly, entries with identical ligands are always present in the same split. When these priorities are in conflict (*i.e.*, identical ligands appear across protein families), the violating entries are removed first from the test set and then from the validation set to maximize training entries. The small- and large-fragment datasets are derived from the initial (all) dataset, but they include only entries with fragments that have ≤3 and ≥4 heavy atoms, respectively. The aromatic, aliphatic, acidic, and basic datasets are derived from the large-fragment dataset, but they include only entries with fragments of the corresponding chemical property. The number of entries (unique PDB IDs) present in each set is reported as an integer, followed by the percent change from the previous step, in parentheses. The relative size of the training, validation, and testing sets at each step (percentages) is given in the rightmost column.

To further ensure the independence of the splits, we next identified entries in different splits whose associated ligands had identical SMILES strings. This comparison required us to first remove those rare Binding MOAD ligands (*e.g.*, peptides) from the validation and testing sets that did not include SMILES strings (Table 1, row 2). We then used the remaining SMILES strings to identify and discard all ligands in the validation and testing sets that shared a ligand in common with any training-set example (Table 1, row 3). Similarly, we discarded all examples remaining in the testing set that shared a ligand in common with any validation-set example (Table 1, row 4).

We next considered the ligand fragments. As our goal was to train DeepFrag to predict fragment additions of modest size (*i.e.*, neither too big nor too small), we eliminated those fragments that had molecular weights greater than 150 Da (Fig. 1C and D) or that contained no heavy atoms (*i.e.*, a single hydrogen atom was not considered a fragment). Any of the entries that were no longer associated with any fragments after this filtering step were removed from the respective sets (Table 1, row 5).

To further focus the dataset on fragments that actually interact with their respective protein receptors, we also eliminated fragments that had no atoms within 4.0 Å of any receptor atom (Table 1, row 6; Fig. 1F). We note that this distance criterion differs from that used in the original DeepFrag model, which instead considered the distance between the trimmed-ligand branching atom (*i.e.*, the atom to which the fragment is connected) and the receptor. See the original DeepFrag article^2^ for full dataset-preparation details.

##### Datasets based on fragment size

To create a small-fragment dataset, we started with the initial (all) dataset but retained only the fragments with three or fewer heavy atoms (≤3 heavy atoms). Any entries that were no longer associated with fragments after this filter step were removed from the dataset entirely (Table 1, row 7a). Similarly, to create a large-fragment dataset, we retained only fragments with four or more heavy atoms (≥4 heavy atoms; Table 1, row 7b). We note that even these “large” fragments are relatively small due to the 150 Da molecular-weight cutoff.

##### Datasets based on chemical properties

To create an aromatic-fragment dataset, we started with the large-fragment dataset but retained only aromatic fragments. Entries no longer associated with any fragments after this filter step were removed from the dataset. We created aliphatic, acidic, and basic datasets following the same protocol.

A fragment was labeled aromatic if it contained any aromatic group; otherwise, it was labeled aliphatic. A fragment was labeled acidic if it contained (1) a carboxylate group, (2) an aryl alcohol or thiol bound to an aromatic carbon atom, (3) a phosphorus or sulfur atom double bonded to an oxygen and singly bonded to an alcohol (*e.g.*, some organic phosphates, phosphonates, sulfates, and sulfonates), (4) a tetrazole, or (5) a sulfur atom double bonded to an oxygen and singly bonded to a nitrogen (*e.g.*, sulfonamides and sulfinamides). On the other hand, a fragment was labeled basic if it contained (1) a primary, secondary, or tertiary aliphatic amine, or (2) an aliphatic nitrogen double-bonded to an atom that is neither an oxygen nor a nitrogen (*e.g.*, many imines, amidines, guanidines, *etc.*). Importantly, some fragments had both basic and acidic moieties. To avoid confusion, we excluded any such examples when preparing datasets for the acidic- and basic-targeted models.

#### Processing receptor/trimmed-ligand/fragment entries

For each entry in the training/validation/testing sets, we voxelated the protein receptor and trimmed ligand, following the same general protocol as in the original DeepFrag publication.^2^ In brief, the atoms of the protein and the trimmed ligand were mapped to grids of 24 × 24 × 24 discrete points spaced 0.75 Å apart, centered on the trimmed-ligand branching atom. A given atom contributed to the grid points that were within 1.75*r* of its center (where *r* is the corresponding van der Waals radius), per an exponential decay function (*i.e.*, the same SMOOTH-2 function used in the original DeepFrag publication^2^). We note that the current version of DeepFrag again differs slightly from the previous version, which assigned the same radius to all atoms, regardless of element.

We calculated separate grids for receptor carbon, oxygen, nitrogen, and sulfur atoms. Other receptor heavy atoms were mapped to a catch-all “other” grid. Similarly, we calculated grids for trimmed-ligand carbon, oxygen, nitrogen, and “other” heavy atoms. These receptor and trimmed-ligand grids were concatenated to yield a single tensor representation of the protein/trimmed-ligand complex, which serves as the model input. In contrast, the cut fragments were not voxelated; rather, they were converted to 2048 bit binary RDKfingerprints (max path: 10)^6,7^ to serve as the model training targets (output). See the SI for a brief discussion of RDKfingerprint performance relative to other common fragment fingerprints (Table S1).

#### Model training

We trained the fragment-specific DeepFrag models using hyperparameters similar to those of the original DeepFrag^2^ (*i.e.*, Adam optimizer; learning rate, 0.0001; batch size, 16; precision, 32). The architecture was also similar to that of the original DeepFrag.^2^ In brief, the model accepts as input a tensor of size [16, 9, 24, 24, 24], where 16 is the batch size, 9 is the number of channels (atom types), and [24, 24, 24] corresponds to the size of each voxel grid. The model processes this tensor in several stages:

1. To normalize the input data, we first apply a batch normalization layer.

2. To detect low-level geometric/chemical features, we apply three 3D convolutional layers (kernel size: 3, no padding), each followed by a ReLU activation layer to introduce non-linearity (*i.e.*, three Conv–ReLU pairs). These three Conv–ReLU pairs successively reduce the tensor size to [16, 64, 22, 22, 22], [16, 64, 20, 20, 20], and [16, 64, 18, 18, 18].

3. To further increase the receptive field, we apply a 3D max pooling layer (kernel size: 2) followed by another Batch Normalization layer, reducing the tensor size to [16, 64, 9, 9, 9].

4. To detect high-level (abstract) representations of the trimmed-ligand/protein complex, we next apply another three Conv–ReLU pairs, successively reducing the tensor size to [16, 64, 7, 7, 7], [16, 64, 5, 5, 5], and [16, 64, 3, 3, 3].

5. To aggregate the 3D spatial information, we next apply global average pooling, reducing the tensor to [16, 64, 1, 1, 1], followed by flattening, producing a tensor of size [16, 64].

6. To project each example into a 512-dimensional embedding space (*i.e.*, the latent representation of the optimal fragment fingerprint), we next apply a dropout layer (rate: 0.5) to prevent overfitting, a fully connected layer to map the [16, 64] tensor to [16, 512], and a ReLU activation layer.

7. Finally, to project the [16, 512] latent-space representation into RDKfingerprint space, we again apply a dropout layer (rate: 0.5) followed by a fully connected layer to map the representation to [16, 2048]. Finally, a sigmoid activation layer ensures the output values fall between 0 and 1, as appropriate for binary fingerprint prediction.

The loss function used during training was calculated as the aggregated cosine distance (1 – cosine similarity) between the DeepFrag-predicted RDKfingerprints (continuous vectors) and the ground-truth RDKfingerprints of the corresponding known fragments (binary vectors). To avoid overfitting and ensure the models learned to make rotation-invariant predictions, we randomly rotated the protein/trimmed-ligand tensors each training epoch. We trained the models on the examples in the training set for 60 epochs.

At the end of each epoch, we calculated the loss on the withheld data of the validation set. To select a “production” model for subsequent analyses, we identified the epoch with the lowest validation-set loss (not necessarily the last epoch) and used the saved model associated with that epoch.

### Protein-specific fine-tuned DeepFrag2 models

#### Dataset preparation

To generate appropriate datasets for the protein-specific fine-tuned DeepFrag models (*i.e.*, models fine-tuned from the general DeepFrag model to improve accuracy on specific protein receptor classes), we first identified the largest protein families cataloged in the Binding MOAD 2020 dataset. Among these, carbonic anhydrase 2 (UniProt accession P00918), beta-secretase 1 (UniProt accession P56817), glutamate receptors (UniProt accessions P19491, P19492, P19493 for rat receptors 2, 3, and 4, and P42262 for human receptor 2), and HIV-1 protease (P03367, P03366, P03369, P04587, P04585, P12499, Q7SSI0, P35963, Q90K99, Q8Q3H0, O92139, Q903J0, Q5RZ08, Q000H7, O09893, Q9Q2G8, P24740, O38723, O38710, O12158, Q9WFL7, Q9Q288, Q9J2R0, Q9J2P7, Q90EB9, Q7ZCL6, Q7SPG9, Q6BB74, Q5RZ09, Q1G1C3, P12499, P0C6F2, P05959, O92103, O38731, O38719, O38708, and O38707) were prominent.

We downloaded the corresponding PDB files for these four classes (622, 452, 481, and 514 structures for carbonic anhydrase 2, beta-secretase 1, glutamate receptors, and HIV-1 protease, respectively). We then separated the protein and ligand structures and fragmented the ligands following the protocol above to generate examples comprised of (protein, trimmed-ligand, fragment) tuples. We again discarded examples with fragments that had (1) molecular weights greater than 150 Daltons or (2) no atoms within 4.0 Å of any receptor atom. We further discarded fragments that had three or fewer heavy atoms. The protein/trimmed-ligand structures were again voxelated, and the fragments were converted to RDKfingerprints.

#### Creating training/validation/testing sets

We adopted a different method for dividing these protein-specific datasets into training, validation, and testing sets. Because the datasets for fine-tuning contained only highly similar (or identical) proteins, we could not initially partition the data by protein family. Instead, we first grouped the data by ligand, using the Butina algorithm^8^ to generate clusters of similar ligands (Tanimoto cutoff of 0.4, applied to Morgan Fingerprints^9^). We then randomly assigned 60% of the clusters to the training set, 20% to the validation set, and 20% to the testing set.

#### Model training

We trained these models for 30 epochs on the examples in their respective training sets, using the same model architecture, hyperparameters, and loss function described above. The approach differed, however, in that the initial weights were taken from the final weights of a previously trained foundational model (*i.e.*, the model trained to predict fragments of all chemical properties with four or more heavy atoms).

#### Codebase refactoring and modernization

To support the development of these new DeepFrag models and improve long-term maintainability, we created a new PyTorch-Lightning-based DeepFrag2 codebase that differs substantially from the original DeepFrag codebase. The new codebase aims to improve modularity and enable easy extension to new model types and data sources. We also added comprehensive caching mechanisms for molecular property calculations and fragment analysis, as well as support for flexible fragment filtering (*e.g.*, by atom-count or chemical property). Beyond enabling the models presented in this work, this modernized DeepFrag2 codebase provides a robust foundation for future extensions to the DeepFrag methodology.

## Results and discussion

This study describes new machine-learning lead-optimization models based on our DeepFrag approach.^1,2^ Like the original DeepFrag model, these new models accept as input the structure of a protein receptor, a bound small-molecule ligand, and the location of a ligand atom where an optimizing terminal fragment should be added. As output, each model predicts an RDKfingerprint-like vector^6,7^ that encodes the topology of a potential optimizing fragment. DeepFrag2 then uses a look-up table (label set) to identify similar fragments with known structures and precalculated fingerprints.

Though the model architecture and evaluation approach are similar, these new models differ from the original DeepFrag implementation in the data used for training, validation, and testing. The original DeepFrag model leveraged the entire Binding MOAD database with its many diverse protein/ligand complexes.^3–5^ In contrast, our new models are more narrowly focused (targeted) to enhance accuracy. Specifically, we test the impact of training models tailored to predict (1) fragments with specific physicochemical properties and (2) fragments suited to specific receptor classes.

### Fragment-specific (targeted) DeepFrag models

#### A new split strategy

To train targeted DeepFrag models tailored to predict fragments with specific sizes or chemical properties, we considered a new approach to splitting the dataset into training, validation, and testing sets. When training on Binding-MOAD data, DeepFrag ensures independence by initially dividing proteins by Binding-MOAD family, such that proteins from the same family are not assigned to different sets. However, independence in ligand identity is also critical to ensure that the model's predictions can generalize (*i.e.*, no ligand that appears in one set should be present in any other).

The original DeepFrag implementation^2^ handled each overlapping-ligand example by randomly selecting one of the overlapping sets to privilege (*e.g.*, the training, validation, or testing set) and removing redundant examples from all others. The main advantage of this strategy is that the relative split sizes remain close to the user-specified proportions. However, fewer examples are available for training because some training examples are discarded. Further, one can imagine that in this scheme, common ligands that are similar (though not identical) might often be placed in different sets (*e.g.*, depending on the random assignments, the training, validation, and testing sets could include GMP-bound, cyclic-GMP-bound, and GDP-bound complexes, respectively).

To address these disadvantages, we used a more rigorous splitting strategy in the present work, which we call the high_priority split scheme. In this scheme, instead of randomly choosing a split to privilege when eliminating a ligand-identical example, the training set is always privileged over the validation and testing sets (*i.e.*, redundant examples present in the training set are always retained in that set and removed from all others). Similarly, the validation set is always privileged over the testing set. Consequently, the proportion of the data allocated to the training and validation sets typically exceeds the user-specified fractions, but more data is available for training. Further, common ligands are less likely to be placed in the testing set, reducing data leakage such as in the GMP/cyclic-GMP/GDP scenario described above. This improved independence reduces the values of Top-*K* metrics as compared to the original DeepFrag paper,^2^ but we believe the resulting models are more generalizable.

To further reduce the chance of data leakage, we considered discarding examples based on compound similarity rather than strict identity. However, enforcing independence in terms of ligand identity already reduced the validation set from 9557 to 5113 structures and the test set from 7581 to 3081 structures (Table 1). Ensuring independence in terms of ligand similarity would have required us to discard even more data. We therefore chose ligand-identity-based filtering to strike a balance between split independence and data retention.

#### Training on the entire Binding MOAD for reference

We initially trained a reference DeepFrag model using examples derived from the entire Binding MOAD, regardless of fragment-size class or chemical property. We call this the “general model” because it was trained on the entire Binding MOAD to predict fragments of any physicochemical class. This model is comparable to the original DeepFrag implementation,^2^ with some important caveats. First, the new model leveraged the Binding MOAD 2020, which contains 41 359 protein-ligand structures, rather than the Binding MOAD 2019 (38 702 structures). Second, we used the new high_priority split scheme to ensure the independence of our training, validation, and testing sets. Use of this scheme produced a training set derived from 22 707 protein/ligand structures (462 103 protein/trimmed-ligand/fragment examples), a validation set derived from 4854 structures (75 220 protein/trimmed-ligand/fragment examples), and a testing set derived from 2890 structures (45 700 protein/trimmed-ligand/fragment examples; Table 2). The fragments included in the training, testing, and validation sets had an average molecular weight of 49 ± 39 Daltons (stdev) and an average heavy atom count of 3.1 ± 2.7 (max: 11).

**Table 2:** Performance comparison of targeted and general DeepFrag models for various fragment types^a^

Fragments	Train set	Val set	Test set	Targeted		General		Random	
Type (Size)	Strucs (Examples)	Strucs (Examples)	Strucs (Examples)	T1	T8	T1	T8	T1	T8
All (any)	22 707 (462 103)	4854 (75 220)	2890 (45 700)	N/A	N/A	44.4	56.1	0.04	0.3
All (≤3)	22 393 (299 139)	4739 (43 002)	2807 (26 961)	69.9	86.2	67.1	82.2	1.3	10.1
All (≥4)	19 824 (162 964)	4349 (32 218)	2591 (18 739)	17.5	30.2	16.3	28.6	0.04	0.3
Aromatic (≥4)	12 367 (45 150)	2987 (12 659)	1738 (6817)	14.4	25.1	11.2	19.1	0.1	0.8
Aliphatic (≥4)	16 276 (117 814)	3155 (19 559)	1925 (11 922)	23.6	41.6	20.7	37.1	0.07	0.6
Acid (≥4)	10 638 (52 492)	1340 (5408)	789 (3812)	50.0	64.7	44.4	61.8	0.4	3.0
Base (≥4)	3021 (14 944)	961 (4472)	480 (1882)	20.1	37.9	13.9	30.0	0.3	2.2

a “Fragments” indicates the chemical properties (“Type”) and heavy atom counts (“Size”) of the fragments used to train the corresponding model. “Strucs” refers to the number of protein-ligand structures, and “Examples” refers to the number of protein/trimmed-ligand/fragment examples derived from those structures. The targeted models were trained only on examples whose fragments had the properties described in the “Fragments” column. The general model was trained on all example types regardless of fragment properties. “Random” indicates a random baseline (*i.e.*, the results obtained when selecting compounds at random from the corresponding test-set-derived label set). Top-*K* values (*K* = 1, 8) represent the percentage of cases where the correct fragment was among the top *K* predictions when the label set comprised all the fragments of the respective targeted (property-specific) testing set. See Table S2 for the Top-16, Top-32, and Top-64 metrics for all models.

We trained the model for 60 epochs on the examples of the training set, saving model checkpoints at each epoch and evaluating each on the independent examples of the validation set. We then selected the model with the lowest validation-set loss as the final DeepFrag model.

To evaluate the performance of this model on the withheld testing set, we constructed a label set of known fragments with precalculated RDKFingerprints from which DeepFrag could select. This label set included fragments derived solely from the ligands of the testing set itself, thus ensuring that the label set always included the ground-truth fragments. One advantage of the DeepFrag approach is that the label set can be entirely independent of the data used for training, validation, and testing. That said, by carefully constructing a label set that includes fragments known to be “correct,” one can evaluate how often DeepFrag selects the “correct” fragment.

For each testing-set example, we ran DeepFrag eight times (each time rotating the protein/ligand complex randomly) and averaged the resulting DeepFrag-predicted RDKfingerprints across these eight rotations. Each averaged fingerprint was subsequently used to select the most similar fragments from the label set. We then calculated the average Top-*K* accuracy across all testing-set examples, where Top-*K* is a metric that indicates how often the ground-truth fragment is among the *K* label-set fragments with fingerprints most similar to DeepFrag's prediction.^2^ We used this same label-set construction scheme and Top-*K* metric to evaluate all models described in this work.

We note that Top-*K* accuracy has several notable limitations that likely lead it to undersell DeepFrag2 performance. For example, the ground-truth fragment is certainly not the single most optimal fragment in all (or even most) examples; in some cases, DeepFrag2 may predict a fragment addition than is better than ground truth, but such a prediction would be counted as “wrong.” Further, Top-*K* accuracy treats all “incorrect” predictions as equally bad, even though predictions that are chemically similar to ground truth should arguably not be as penalized. See the SI for further discussion of these strengths and weaknesses. That said, despite these limitations, we prefer Top-*K* for assessing model accuracy because it is an easy-to-interpret and easy-to-calculate metric.

When we evaluated the general model on its withheld testing set, we found that the testing-set fragment most similar to the DeepFrag prediction (*i.e.*, Top-1 accuracy) was the correct fragment 44.4% of the time. The correct fragment was among the eight most similar fragments (*i.e.*, Top-8 accuracy) 56.1% of the time. In contrast, we would have expected Top-1 and Top-8 accuracies of 0.04% and 0.3%, respectively, had we simply selected at random from the 2453 fragments in the test-set-derived label set (Tables 2 and S2).

#### Predicting fragments of different size classes

Notably, the accuracy of the general DeepFrag2 model is higher for smaller fragments (*e.g.*, hydroxyl or methyl moieties). We hypothesize that this improved accuracy on smaller fragments results from two factors. First, smaller fragments are more prevalent in the training data, comprising approximately 63% of all examples (Table 2). Consequently, the general model must allocate substantial capacity to learning distinctions among these abundant small fragments, potentially limiting its ability to accurately predict the less prevalent larger fragments. Second, smaller fragments have a more constrained chemical space that makes accurate prediction easier. There are only so many unique small fragments possible; in contrast, the number of possible larger fragments expands dramatically with increasing heavy-atom count, making it far more challenging to select the correct answer from a much larger pool of possibilities.

To demonstrate this bias in favor of small fragments, we separately applied the general model (trained on fragments of all sizes) to the small and large fragments of its corresponding testing set, in each case using the predicted RDKfingerprints to select from a label set comprised of testing-set fragments with the correspondingly appropriate sizes. When we considered only the small-fragment examples, we found the Top-8 accuracy was 82.2%, far higher than the accuracy obtained when the general model was applied to and evaluated on fragments of all sizes (56.1%, see above). When we considered only the large-fragment examples, we found the Top-8 accuracy was only 28.6% (Table 2).

To address this apparent bias, we developed separate DeepFrag models specifically trained to predict small fragments (≤3 heavy atoms) and large fragments (≥4 heavy atoms). The fragments included in the small-fragment training, testing, and validation sets had an average molecular weight of 23 ± 12 Daltons (standard deviation) and an average heavy atom count of 1.4 ± 0.7 (max: 3). In contrast, the fragments included in the large-fragment training, testing, and validation sets had an average molecular weight of 97 ± 26 Daltons and an average heavy atom count of 6.4 ± 2.0 (min: 4; max: 11).

Both the small- and large-fragment models showed improved accuracy within their respective domains. When we applied the small-fragment model to its testing set (using the same small-fragment testing-set examples as a label set), the Top-8 accuracy increased from 82.2% (general model) to 86.2%. Similarly, the Top-8 accuracy for large-fragment predictions increased from 28.6% (general model) to 30.2% when using the large-fragment model. Notably, we would have expected Top-8 accuracies of 10.1% and 0.3%, respectively, had we randomly selected from the 79 or 2374 fragments in the corresponding test-set-derived label sets (Tables 2 and S2).

#### Predicting fragments with specific chemical properties

We also trained separate DeepFrag models on large fragments (≥4 heavy atoms) that are (1) aromatic, (2) aliphatic, (3) acidic, or (4) basic. Our motivation for training these models is twofold. First, we recognize that medicinal chemists can often guess the properties of a suitable fragment, even if they cannot immediately identify an exact fragment structure. For example, if a receptor binding pocket includes a basic residue such as arginine, one might reasonably suppose that the corresponding moiety on the ligand should be acidic (*e.g.*, a carboxylate). Second, because the general model must allocate capacity to learning distinctions among all fragment types, its performance on any specific chemical class may be diluted. Targeted models focus entirely on their specific domain and so should have improved accuracy within their respective classes.

To create such models, we filtered the large-fragment (≥4 heavy atoms) training, validation, and testing sets to include only fragments with the appropriate chemical properties for each targeted model. The fragments included in the basic, acidic, and aliphatic training/testing/validation sets had comparable sizes (molecular weights: 95 ± 27, 93 ± 21, and 92 ± 26 Daltons, respectively; heavy atom counts: 6.5 ± 2.0, 5.4 ± 1.7, and 5.8 ± 1.8, respectively, ranging from 4 to 11 heavy atoms in all cases). The fragments included in the aromatic training/testing/validation sets were somewhat larger (molecular weight: 110 ± 23 Daltons; heavy atom count: 8.0 ± 1.6, ranging from 5 to 11 heavy atoms).

Each of the chemical-property targeted models trained and evaluated on these sets had an improved Top-8 accuracy over the general model when applied to the same targeted testing set, using the corresponding testing-set-associated fragments as a label set (Table 2). The impact when predicting basic fragments was particularly notable; Top-8 accuracy increased from 30.0% (general model) to 37.9% (targeted model).

#### Consistency of physicochemical property predictions

Having shown that DeepFrag models trained on fragments with specific physicochemical properties generally have improved Top-*K* accuracies, we next assessed whether those models had truly learned to select fragments with the targeted properties, regardless of the label set chosen. To calculate the Top-*K* accuracy metrics above, each targeted model was allowed to select fragments from a label set derived from the corresponding test set, which consisted exclusively of fragments with the corresponding target property (*e.g.*, the label set used to evaluate the basic-fragment model included only basic fragments). Consequently, though the targeted models could select wrong fragments in terms of chemical identity, they could not select wrong fragments in terms of chemical property. We chose this evaluation paradigm because it best mimics real-world use; if medicinal chemists suspect a basic fragment is appropriate, they should select both a basic-targeted model and a basic-targeted label set.

That said, we hypothesized that if our targeted models had truly learned to select fragments with specific physicochemical properties, they should select such fragments even when other fragment types are included in the label set, and even when the ground-truth fragment itself does not have the targeted property. To test this hypothesis, we applied the trained small-fragment, large-fragment, aromatic, aliphatic, acidic, and basic models to the examples in the general-model testing set, which contains fragments spanning all size and chemical classes. We used these same physicochemically diverse testing-set fragments as the label set for selection. Importantly, because of the consistent split strategy used across all models, none of the general-model testing-set examples were included in the targeted-model training or validation sets, ensuring continued independence.

For each model, we considered only the Top-1 selected fragment from the label set whose fingerprint was most similar to DeepFrag's predicted fingerprint. We then assessed how often this Top-1 fragment had the expected physicochemical property. Even though the targeted models could, in this case, select fragments of any chemical size and type, they still overwhelmingly selected fragments with the correspondingly appropriate property (Table 3), demonstrating that the targeted models have learned to bias their fragment selections appropriately.

**Table 3:** Property-specific bias of targeted DeepFrag models^a^

	All (≤3)	All (≥4)	Aromatic	Aliphatic	Acid	Base
Top-1 property match (%)	100.00%	98.32%	100.00%	99.69%	97.74%	98.28%

a Percentage of cases where targeted DeepFrag models selected fragments with the expected physicochemical property. Each targeted model was evaluated on the general-model testing set (containing fragments of all sizes and chemical properties) using the same diverse fragments as the label set. The “Top-1 Property Match (%)” indicates how often the top-ranked selected fragment possessed the target property that the model was trained to predict, demonstrating that targeted models maintain their property-specific biases even when allowed to choose from fragments spanning all size and chemical classes.

#### Accuracy of specific fragment predictions as a function of cosine similarity

We expect many DeepFrag users will ask the question, “Given a specific DeepFrag-selected fragment of interest, what are the chances that fragment will be useful?” To this end, we examined whether the cosine-similarity score associated with a given fragment can serve as a reliability indicator for that fragment's usefulness. If so, medicinal chemists could use similarity scores to evaluate whether a specific fragment is worth pursuing *via* synthesis and experimental testing.

**Fig. 2:**
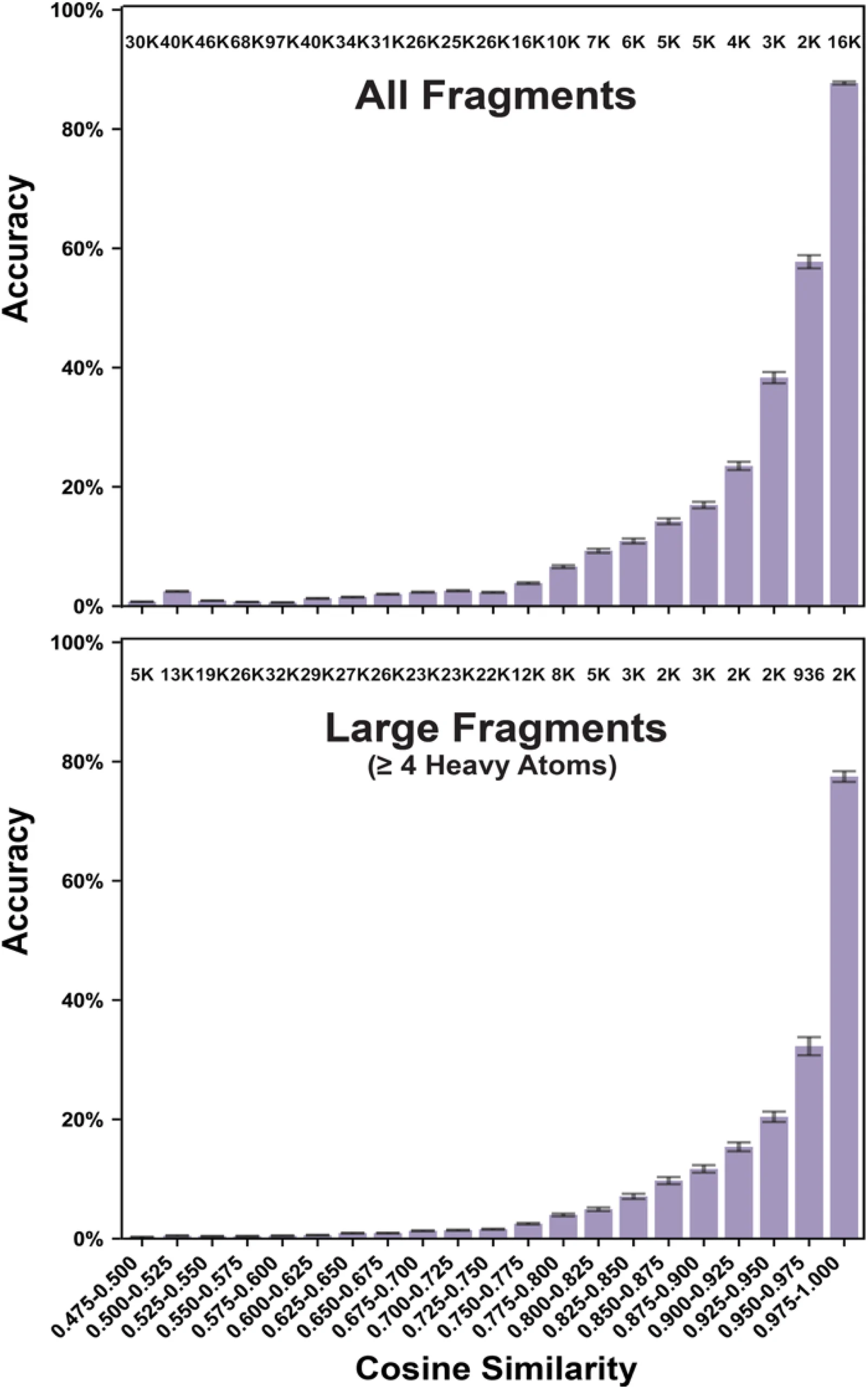
Relationship between a fragment's cosine-similarity score and its likelihood of matching the ground-truth fragment, regardless of rank. The plots show accuracy (purple bars) binned by cosine similarity, where accuracy represents the percentage of cases where the selected fragment matched the ground-truth fragment within each bin. Source data includes all Top-16 fragment selections from all corresponding testing-set examples. The upper panel shows results from the general model (trained on fragments of all chemical properties and sizes), and the lower panel shows results from the large-fragment model (≥4 heavy atoms). In both cases, the label set was comprised of all fragments from the corresponding testing set. Numbers above each bin indicate the sample size, abbreviated where necessary (*K* represents thousands). Error bars represent standard error. See the SI (Fig. S8–S12) for similar graphs corresponding to the small-fragment, basic, acidic, aromatic, and aliphatic models, as well as an alternative analysis for the interested reader.

For each testing-set example, we examined the Top-16 selected fragments from the label set and calculated the cosine similarity between each fragment's fingerprint and the corresponding DeepFrag-predicted fingerprint. We then binned all these fragment predictions by their cosine similarity values and calculated the accuracy within each bin (Fig. 2, purple bars). In other words, for each cosine-similarity bin, we determined the percentage of cases in which the selected fragment matched the ground-truth fragment.

Our results revealed a strong relationship between high cosine similarity and increased accuracy. Fragments with cosine similarity scores above 0.975 showed markedly higher accuracy rates compared to those in lower similarity bins, with accuracy generally declining as cosine similarity decreased. This finding suggests that cosine similarity alone is indeed a useful metric for evaluating the reliability of specific fragment recommendations.

That said, in practice, users may wish to select a lower threshold. Fragments with somewhat lower cosine-similarity scores could nevertheless be structurally and chemically relevant bioisosteres, making them effective fragment additions.

#### Protein-specific fine-tuned DeepFrag models

We also explored the impact of fine-tuning DeepFrag models on specific protein-receptor classes. Our goal was to adapt the broad chemical and structural insights captured by a foundational model to the specific characteristics of individual protein families. As proof of principle, we applied our fine-tuning protocol to the beta-secretase 1, glutamate receptor, HIV-1 protease, and carbonic anhydrase 2 protein families.

In all cases, we started fine-tuning from the large-fragment foundational model, which had been previously trained for 60 epochs on examples with fragments of at least four heavy atoms, derived from the entire Binding MOAD dataset. Fine-tuning proceeded for 30 epochs using only protein/ligand complexes from the respective protein family, maintaining the four-heavy-atom constraint.

To evaluate the performance of these receptor-class-specific fine-tuned models, we saved model checkpoints every epoch and calculated Top-*K* metrics on the testing set per epoch, as described above. This approach allowed us to monitor how additional training continuously improved performance on each protein class over the course of the 30-epoch run.

**Fig. 3:**
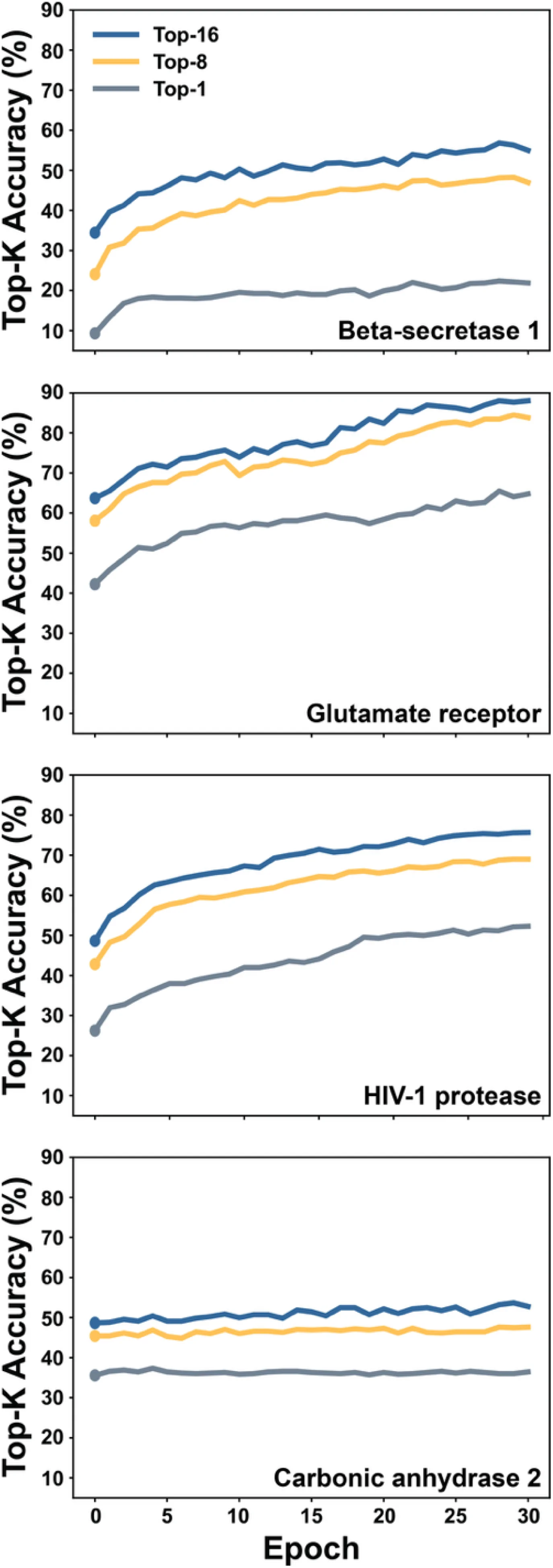
Performance of DeepFrag models finetuned on four specific protein systems: beta-secretase 1, the glutamate receptor, HIV-1 protease, and carbonic anhydrase 2. The graphs show the Top-*K* accuracy (*K* = 1, 8, 16) as a function of training epoch. Top-1, Top-8, and Top-16 accuracies are represented by the bottom (gray), middle (gold), and top (navy blue) lines, respectively.

Critically, the beta-secretase 1, glutamate receptor, and HIV-1 protease protein families were assigned to the validation set of the original large-fragment foundational model, not its training set. Use of these three receptor classes thus allows us to evaluate model improvement when fine-tuning on unseen examples. Generation 0 represents the performance of the foundational model applied to the same examples of the respective receptor-specific testing sets, serving as a baseline before fine-tuning (Fig. 3). Top-8 accuracy (gold lines) generally improved as fine-tuning progressed, particularly in the early epochs (Fig. 3). After 30 epochs of training, the fine-tuned beta-secretase 1, glutamate receptor, and HIV-1 protease models had Top-8 accuracies that were 22.9, 25.7, and 26.2 percentage points higher than those of the foundational model when applied to the same receptor-specific testing set (Table 4; see Fig. S13–S15 for examples of fine-tuned-model predictions, with foundational-model predictions for comparison).

**Table 4:** Performance comparison of fine-tuned and foundational DeepFrag2 models for specific protein classes^a^

Protein	Train set	Val set	Test set	Fine-tuned model (after 30 epochs)			Foundational model (all ≥4, after 60 epochs)		
	Strucs (Examples)	Strucs (Examples)	Strucs (Examples)	T1	T8	T16	T1	T8	T16
Beta-secretase 1	176 (921)	75 (396)	178 (773)	21.9	47.0	55.0	9.3	24.1	34.4
Glutamate receptor	228 (523)	77 (169)	115 (284)	64.8	83.8	88.0	42.3	58.1	63.7
HIV-1 protease	260 (2010)	87 (683)	154 (1175)	52.3	69.0	75.7	26.2	42.8	48.6
Carbonic anhydrase 2	195 (531)	62 (189)	258 (680)	36.5	47.6	52.8	35.6	45.4	48.7

a “Protein” indicates the specific protein class on which the model was fine-tuned. “Strucs” refers to the number of protein-ligand structures, and “Examples” refers to the number of protein/trimmed-ligand/fragment examples derived from those structures. The fine-tuned model was trained only on examples whose fragments had four or more heavy atoms, derived from ligands known to bind members of the corresponding protein class. The foundational model was trained on examples with large fragment of all chemical types. Top-*K* values (*K* = 1, 8, 16) represent the percentage of cases where the correct fragment was among the top *K* predictions when the label set included only fragments from the respective testing set, derived from known ligands.

In contrast, the carbonic anhydrase 2 protein family was assigned to the training set of the large-fragment foundational model. Use of this receptor class thus allows us to evaluate model improvement when fine-tuning on a protein family which which the foundational model is already familiar. Notably, though improvement was not as dramatic, fine-tuning on this dataset did still improve Top-8 accuracy by 2.2 percentage points. See Fig. S16 for examples of fine-tuned-model predictions for this family, with foundational-model predictions for comparison.

These results suggest that fine-tuning can effectively adapt a foundational DeepFrag2 model to specific protein families. These improvements are particularly notable when fine-tuning on proteins not included in the DeepFrag2 training set, but even fine-tuning on known protein families can be useful. We expect that such protein-specific models will be particularly valuable for drug discovery campaigns targeting proteins with many known ligands available for training, especially proteins that were not extensively characterized in the public literature prior to 2020, such as those represented in large proprietary datasets or targets of more recent therapeutic interest.

#### DeepFrag2 in the context of other machine-learning models for structure-aware lead optimization

Machine-learning approaches have fundamentally transformed many areas of drug discovery. Among these approaches are others that, like DeepFrag2, leverage the three-dimensional structure of the protein binding pocket to guide lead optimization. Many of these structure-aware models address lead-optimization challenges that are complementary to but distinct from DeepFrag2's fragment-addition task. For example, some tools guide fragment linking, a lead-optimization strategy that aims to create improved ligands by connecting two or more molecular fragments known to bind in different sub-pockets. Recent advances in this area include diffusion models such as DiffLinker,^10^ which can design appropriate chemical linkers while respecting the geometric constraints of the protein binding site. Still other machine-learning tools guide scaffold hopping, a strategy that involves replacing a lead compound's core structure while preserving the orientation of its key side chains to maintain critical interactions with the receptor. Recent deep-learning approaches such as DiffHopp^11^ and the faster TurboHopp^12^ address this challenge using conditional graph diffusion or consistency models to generate novel scaffolds that fit within the target's binding pocket.

Still other structure-aware tools perform the same fundamental task as DeepFrag2: modifying decorating moieties about a core molecular scaffold. One common approach for optimizing such moieties uses an evolutionary algorithm to iteratively grow molecules from a starting scaffold. The LigBuilder series of tools, for example, builds ligands step-by-step from a fragment library.^13–15^ Similarly, our AutoGrow series uses a genetic algorithm to evolve optimized decorations through repeated cycles of fragment addition/swapping and fitness evaluation *via* molecular docking.^16–18^

More recently, several generative deep-learning methods have emerged to address this same challenge of optimizing decorating moieties conditioned on the 3D pocket environment. These approaches include DiffDec,^19^ a graph neural network and diffusion model designed to generate structure-aware moieties. Similarly, Diffleop guides the generative process using predicted binding affinity gradients to produce more potent analogs.^20^ In contrast, Lingo3DMol uses language models, geometric deep learning, and a specialized 3D-aware molecular representation to suggest modifications that properly fit the binding site.^21^ Particularly notable is the recently published Delete approach, which leverages a versatile masking strategy to train a single generative model that can handle fragment growing, linking, and replacement.^22^

That said, DeepFrag2 occupies a distinctive position within this emerging field. Its methodology differs fundamentally from the *de novo* approaches of the generative models. Instead of directly generating novel chemical structures, DeepFrag2 predicts a target chemical fingerprint. Medicinal chemists can then use this fingerprint to screen a pre-existing library of carefully vetted fragments to find the best match. DeepFrag2-selected fragment additions thus depend entirely on the provided fragment library.

This constraint complicates efforts to quantitatively compare DeepFrag2 performance with that of the *de novo* generative approaches. While one could in principle adapt a Top-*K* accuracy metric for generative models (generating *K* candidates and evaluating how often the correct fragment appears), such a comparison would be inherently unfair to the generative methods. DeepFrag2 must select fragments from the provided label set, which, to calculate Top-*K* accuracy, must include the “correct” answers as possible options. Generative approaches are not so constrained; they can suggest any fragment without limitation, including fragments that may not be chemically valid. This greater freedom makes generative models less likely to reproduce a specific “correct” fragment, even when their outputs are chemically reasonable and potentially useful.

Limiting fragment selection to a pre-existing library may restrict DeepFrag2's ability to explore novel chemical space. But this same constraint is practically useful in that it allows medicinal chemists to limit selection to fragments with desired characteristics (*e.g.*, certain physicochemical properties, reasonable synthesizability, existing availability in chemical repositories, *etc.*).

## Conclusion

This work extends the DeepFrag methodology by developing new models with improved accuracy through domain-specific training. We found that training models on fragments with defined physicochemical properties (small *versus* large, aromatic *versus* aliphatic, and acidic *versus* basic) consistently improves prediction accuracy within their respective domains. Further, our protein-specific fine-tuning results show meaningful improvements for three of the four receptor classes tested, suggesting receptor-specific fine-tuning can substantially improve accuracy for focused drug-discovery campaigns.

That said, the present approach does have its limitations. For example, if users have no prior insight into the most suitable sizes or chemical properties of candidate optimizing fragments, the original general-purpose DeepFrag model may be more useful than our fragment-specific models. We especially recommend the general model for researchers who are conducting exploratory optimizations and wish to avoid preconceptions about chemical space, or who are considering complex binding sites with mixed characteristics (*e.g.*, both hydrophobic and polar regions), where multiple fragment properties may be simultaneously important.

We also note that, as a voxel-based approach, DeepFrag2 is not perfectly rotationally invariant (*i.e.*, it can predict different fragment fingerprints at inference depending on how the protein/ligand complex is rotated about the user-specified branching atom). We chose to use a voxel-based approach because of its simplicity and proven effectiveness.^2^ Further, we have found that our current method of randomly rotating each protein/ligand complex every training epoch much improves rotational consistency.^2^ That said, equivariant architectures (*e.g.*, equivariant graph neural networks) certainly represent a promising future direction.

Our fine-tuning approach also has its limitations. Though fine-tuning DeepFrag models can substantially improve accuracy for some receptor classes, it requires extensive computational resources and may not be accessible to the standard user. Fine-tuning is also feasible only when the receptor class of interest already has many known ligands available for training.

These weaknesses aside, we are hopeful that providing medicinal chemists with DeepFrag models tailored to particular fragment classes and target proteins will reduce the time and resources required to identify promising chemical modifications. The DeepFrag source code, trained models, and an easy-to-use Google Colab are available free of charge from https://durrantlab.com/deepfrag2/, released under the open-source MIT License.

## Author contributions

Harrison Green, Shayne D. Wierbowski, and Jacob D. Durrant conceived the research. César R. García-Jacas, Harrison Green, Shayne D. Wierbowski, and Jacob D. Durrant developed the methodology. César R. García-Jacas and Jacob D. Durrant performed the investigation and formal analysis. César R. García-Jacas, Harrison Green, and Jacob D. Durrant developed the software. Jacob D. Durrant curated the data and was responsible for visualization. César R. García-Jacas, Shayne D. Wierbowski, and Jacob D. Durrant performed the validation. Shayne D. Wierbowski and Jacob D. Durrant acquired funding, provided resources, administered the project, and supervised the research. César R. García-Jacas and Jacob D. Durrant wrote the original manuscript draft. All authors contributed to the review and editing of the final manuscript.

## Conflicts of interest

S. D. W. is an employee of Pfizer, Inc. The authors declare that they have no known competing financial interests or personal relationships that could have appeared to influence the work reported in this paper.

## Supplementary Material

DD-005-D5DD00425J-s001

## Data Availability

The code required to train and use DeepFrag2 can be found at https://github.com/durrantlab/deepfrag2. Fragment libraries and trained models can be downloaded from https://durrantlab.pitt.edu/apps/deepfrag2/models/. An archived version of the same files is accessible through Zenodo (https://doi.org/10.5281/zenodo.18626809). Supplementary information (SI) is available. See DOI: https://doi.org/10.1039/d5dd00425j.
