# Supplementary material for: Precision fragment addition: domain-specific DeepFrag2 models for smarter lead optimization
Source: Digit Discov. 2026 Feb 25;5(3):1340–50. doi: 10.1039/d5dd00425j (PMC12935096; doi:10.1039/d5dd00425j)
Supplement: DD-005-D5DD00425J-s001 [file DD-005-D5DD00425J-s001.pdf]

Supplementary Information (SI) for Digital Discovery.
This journal is © The Royal Society of Chemistry 2026

Precision Fragment Addition: Domain-Specific DeepFrag2 Models for Smarter Lead Optimization (Supporting Information)

César R. García-Jacas[1], Harrison Green[1], Shayne D. Wierbowski[2], Jacob D. Durrant[1,*]

[1] Department of Biological Sciences, University of Pittsburgh, Pittsburgh, PA, USA
[2] Machine Learning and Computational Sciences, Pfizer Research and Development, Cambridge, MA, USA
[*] To whom correspondence should be addressed.

Strengths and Weaknesses of Top-K Metric

In the current work, we used Top-K accuracy as our primary evaluation metric because it provides an easy-to-interpret approach for comparing model performance, but the method certainly has its limitations. For example, the "correct" fragment, as defined by the examples in our dataset, is certainly not the absolute optimal choice in all scenarios. We encountered a good illustration of this limitation when testing DeepFrag on the FDA-approved drug oseltamivir bound to influenza neuraminidase (2HU4:A[1]). When we removed the oseltamivir carboxylate and used DeepFrag to predict a suitable replacement, its top selection was a phosphonate group, not the "correct" carboxylate group. And yet, though no crystal structure including oseltamivir phosphonic acid is present in the Protein Data Bank, studies suggest that phosphonate is a suitable bioisostere of carboxylate in this case; indeed, the phosphonic acid form is more active than the "correct" carboxylate form (IC_{50}: 0.52 nM vs. 0.73 nM)[2].

Top-K accuracy is also limited as a metric because it treats all incorrect predictions equally without considering the chemical similarity or biological equivalence of the suggested fragments. In an ideal assessment, predictions that are chemically similar to a ground-truth fragment should not be penalized as much as entirely unrelated predictions. See Figure S1, second row, for an example of a prediction that is technically wrong and yet clearly very similar to the ground truth.

A third limitation pertains specifically to Top-1 accuracy (i.e., how often the most-similar label-set fragment is identical to the ground-truth fragment). In real-world drug discovery, medicinal chemists typically synthesize and evaluate a series of compounds rather than a single (top) candidate. A model's ability to suggest multiple promising fragments is often more valuable than its ability to predict a single "correct" answer. We therefore consider Top-8 accuracy (i.e., how often the ground-truth fragment is among the eight most-similar label-set fragments) to be a more appropriate metric for assessing DeepFrag's performance.

2048-Bit RDKfingerprints Enable Improved Top-K Accuracy

We encoded molecular fragments as 2048-bit RDKfingerprints for DeepFrag2 training and label-set lookup (see main text). To justify this fingerprint size, we calculated 2048-bit RDKfingerprints for all unique fragments in the all-fragment dataset. We found that each of the 2048 bits was occupied in at least some of the fragment fingerprints (min prevalence 3.7%, max prevalence 69.7%, average prevalence 10.2%), demonstrating that no bit went unused.

Further, we found that RDKfingerprints performed slightly better than other molecular fingerprints tested. For the sake of comparison, we trained three versions of the general (all-fragments) model for sixty epochs using 2048-bit RDKfingerprints, 2048-bit circular Morgan fingerprints[3], and 166-bit structural-key MACCS fingerprints[4], respectively, using the same all-fragment training set described in the main text. We then evaluated each last-epoch trained model on the same main-text testing set and calculated Top-K metrics. RDKfingerprint DeepFrag2 consistently had Top-K metrics slightly better than Morgan and MACCS DeepFrag2 (Table S1), though Morgan DeepFrag2 was comparable.

Table S1. Top-K metrics for DeepFrag2 models trained using RDKfingerprints, Morgan fingerprints, and MACCS fingerprints.

**Fingerprint**	**Size**	**Top-1**	**Top-8**	**Top-16**
RDKfingerprint	2048	44.4	56.1	59.0
Morgan	2048	44.0	55.4	58.0
MACCS	166	42.1	52.3	55.3

"Fingerprint" indicates the molecular fingerprint used, and "Size" indicates the length of the fingerprint vector. Each model was trained for 60 epochs on the same all-fragment training set used to create the general model described in the main text. Each final-epoch model was applied to the same general-model testing set, and the testing-set predictions were used to calculate Top-K metrics (K = 1, 8, 16; i.e., the percentage of cases where the correct fragment was among the top *K* predictions).

Complete Report of Top-K Metrics for Each DeepFrag2 Model

In the main text, we report the Top-1 and Top-8 metrics for each trained DeepFrag2 model. Table S2 extends this information to include Top-16, Top-32, and Top-64 metrics.

Table S2. Performance comparison of targeted and general DeepFrag models for various fragment types.

**Fragments**	**Targeted**					**General**					**Random**					**Label-Set Size**
Type (Size)	*T1*	*T8*	*T16*	*T32*	*T64*	*T1*	*T8*	*T16*	*T32*	*T64*	*T1*	*T8*	*T16*	*T32*	*T64*	
all (any)	N/A	N/A	N/A	N/A	N/A	44.4	56.1	59.0	62.3	66.1	0.04	0.3	0.7	1.3	2.6	2,453
all (≤ 3)	69.9	86.2	89.2	92.3	97.9	67.1	82.2	86.5	90.5	97.5	1.3	10.1	20.3	40.5	81.0	79
all (≥ 4)	17.5	30.2	34.0	37.7	42.4	16.3	28.6	32.1	36.3	42.0	0.04	0.3	0.7	1.3	2.7	2,374
aromatic (≥ 4)	14.4	25.1	28.9	32.9	39.5	11.2	19.1	22.9	26.5	32.4	0.1	0.8	1.6	3.3	6.6	975
aliphatic (≥ 4)	23.6	41.6	46.4	52.6	58.8	20.7	37.1	41.8	47.1	54.3	0.1	0.6	1.1	2.3	4.6	1,399
acid (≥ 4)	50.0	64.7	68.8	73.8	79.5	44.4	61.8	66.6	72.7	78.4	0.4	3.0	6.0	12.0	24.0	267
base (≥ 4)	20.1	37.9	49.5	58.0	66.4	13.9	30.0	37.7	46.2	58.8	0.3	2.2	4.4	8.8	17.6	363

"Fragments" indicates the chemical properties ("Type") and heavy atom counts ("Size") of the fragments used to train the corresponding model. "Strucs" refers to the number of protein-ligand structures, and "Examples" refers to the number of protein/trimmed-ligand/fragment examples derived from those structures. The targeted models were trained only on examples whose fragments had the properties described in the "Fragments" column. The general model was trained on all example types regardless of fragment properties. "Random" indicates a random baseline (i.e., the results obtained when selecting compounds at random from the corresponding test-set-derived label set). Label-Set Size indicates the number of unique fragments in the testing-set-derived label set. Top-K values (K = 1, 8, 16, 32, 64) represent the percentage of cases where the correct fragment was among the top *K* predictions when the label set comprised all the fragments of the respective targeted (property-specific) testing set.

Examples of General-Model Performance

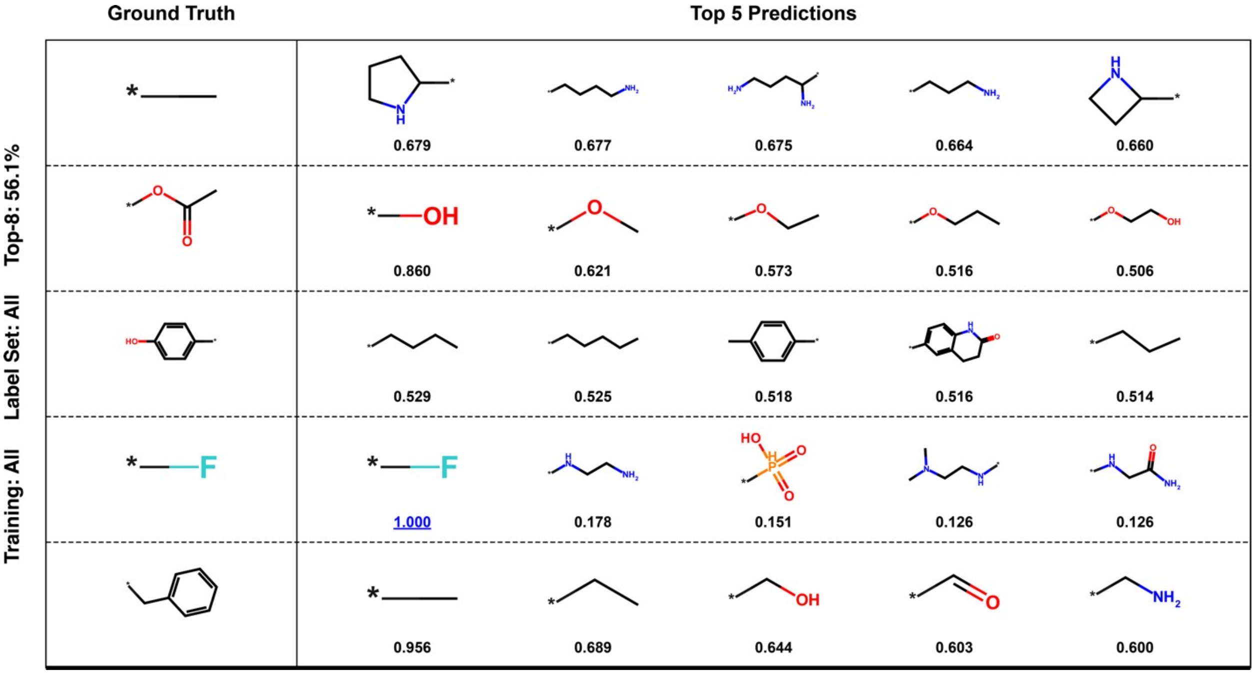

Figure S1. General DeepFrag2 model (trained on fragments of all size classes and chemical properties) applied to five randomly selected testing-set examples. The label set included all testing-set fragments. In many cases, even when DeepFrag2 doesn't select the correct fragment, it selects chemically similar fragments (e.g., row 2).

Binned Top-1 accuracy analysis as a function of cosine similarity

We expect many DeepFrag users will ask, "Given a set of top DeepFrag-selected fragments, what are the chances a useful fragment is among them?" To this end, we considered Top-1 cosine similarity scores, defined as the cosine similarity between the Top-1 (most similar) label-set fragment fingerprint and the corresponding DeepFrag-predicted fingerprint. We examined whether there exists a reasonable Top-1 cosine-similarity threshold above which one can reliably conclude that the ground-truth fragment is among the most similar label-set compounds. If so, medicinal chemists could use Top-1 cosine similarity as a practical confidence metric.

For each model, we first determined the Top-1 cosine-similarity score of each example in the corresponding testing set. We then binned the testing-set examples by their Top-1 cosine similarity scores and calculated the Top-1 accuracy within each bin (Figure S2, purple bars). In other words, for each Top-1 cosine-similarity bin, we determined the percentage of cases in which the ground-truth fragment was in fact the top-ranked selection.

We extended this analysis to include Top-8 and Top-16 accuracy (Figure S2, gray and black lines, respectively). Here we again binned the examples by the same Top-1 cosine similarity scores, but we considered how often the ground-truth fragment was among the Top-8 (and Top-16) most similar fragments from the respective label set.

Our results revealed a strong relationship between high Top-1 cosine similarity and increased Top-1, Top-8, and Top-16 accuracy, with the highest-similarity bins demonstrating exceptional accuracy in many cases (Figures S2-S7). This finding suggests that high Top-1 cosine similarity can

indeed be interpreted as a confidence score, providing a quantitative measure of prediction reliability. If the cosine similarity associated with the Top-1 selected fragment is greater than 0.975, the selected fragment is likely to match the ground-truth fragment.

That said, in practice users may wish to select a lower threshold. Fragments with somewhat lower cosine-similarity scores could nevertheless be structurally and chemically relevant bioisosteres, making them effective fragment additions.

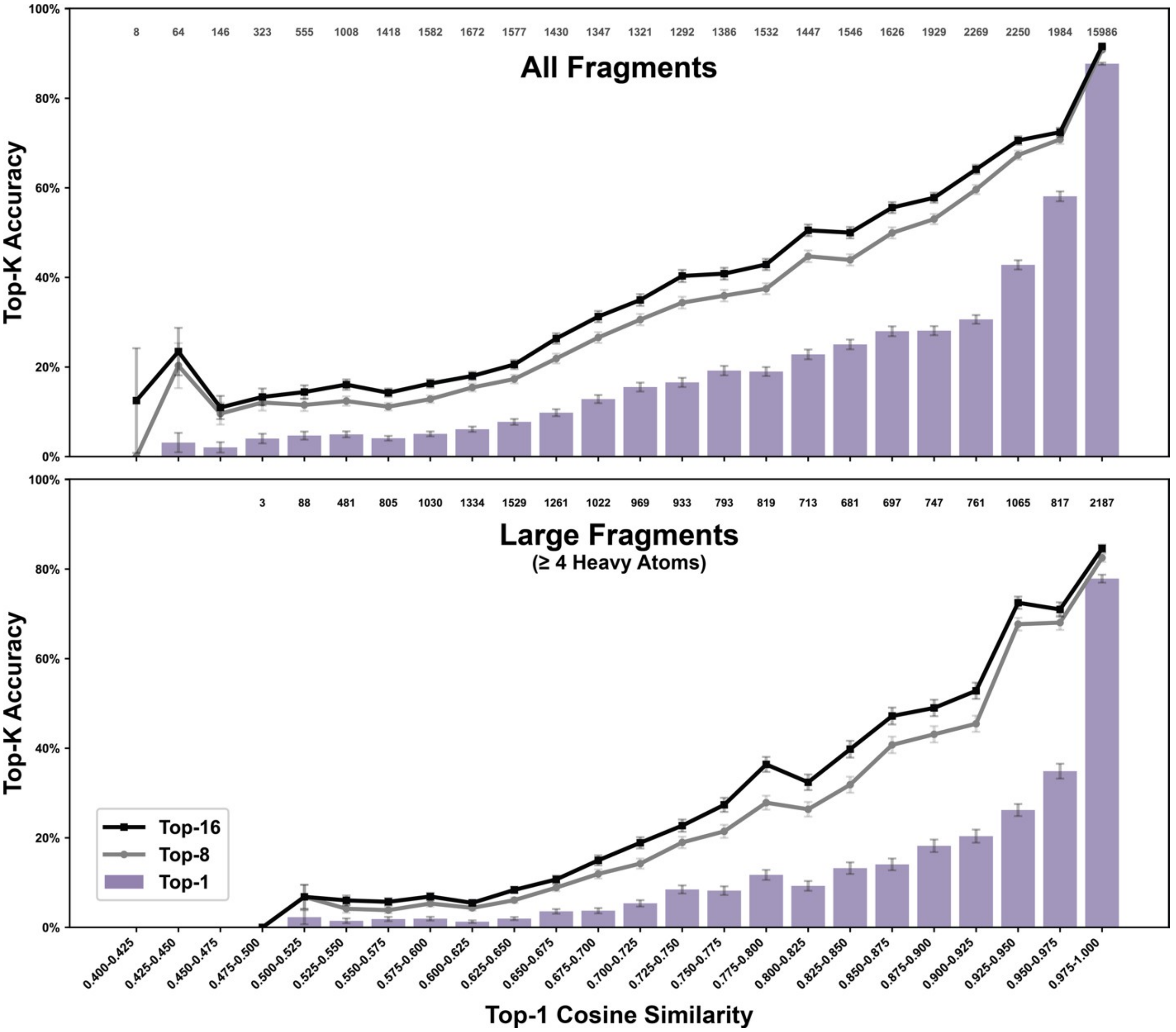

Figure S2. Relationship between Top-1 cosine similarity and Top-K accuracy. The plots show Top-1 (purple bars), Top-8 (gray line), and Top-16 (black line) accuracies binned by Top-1 cosine similarity. The upper panel shows results from the general model (trained on fragments of all chemical properties and size classes), and the lower panel shows results from the large-fragment model (≥ 4 heavy atoms). In both cases, the label set was comprised of all fragments derived from the corresponding testing set. Numbers above each bin indicate the sample size. Error bars represent standard error.

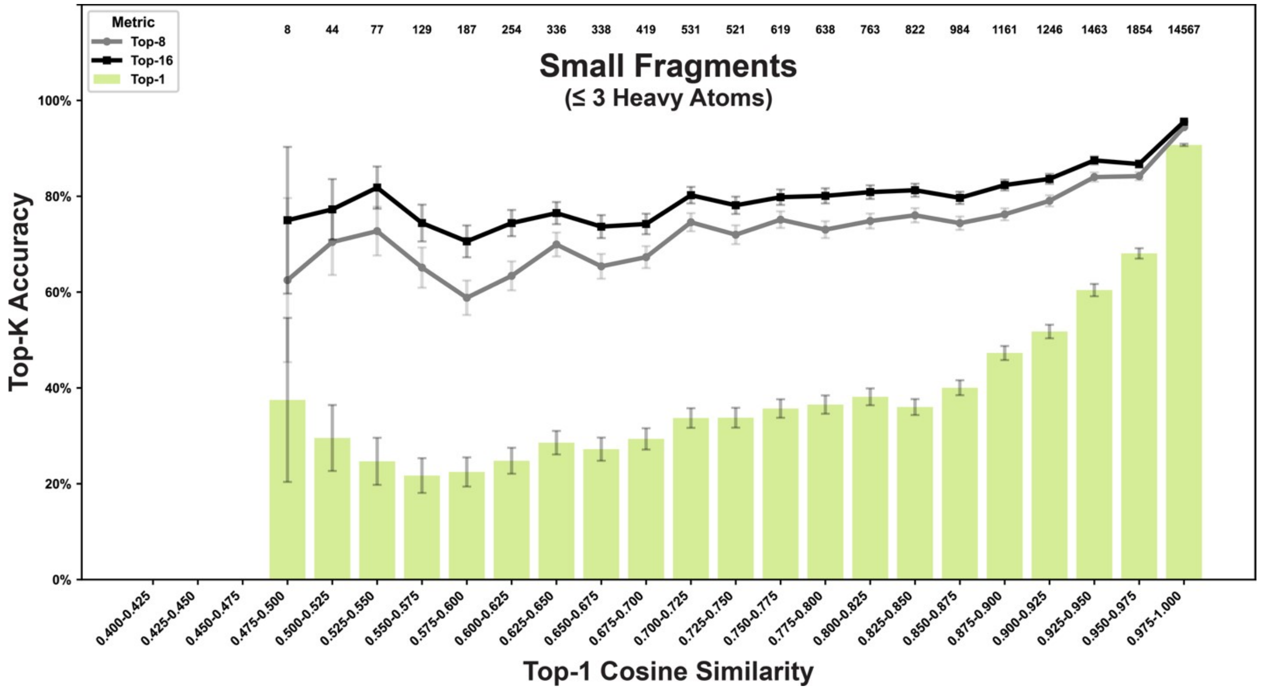

Figure S3. Relationship between Top-1 cosine similarity and Top-K accuracy for the small-fragment model (≤ 3 heavy atoms). The plots show Top-1 (bars), Top-8 (gray line), and Top-16 (black line) accuracies binned by Top-1 cosine similarity. Numbers above each bin indicate the sample size. Error bars represent standard error.

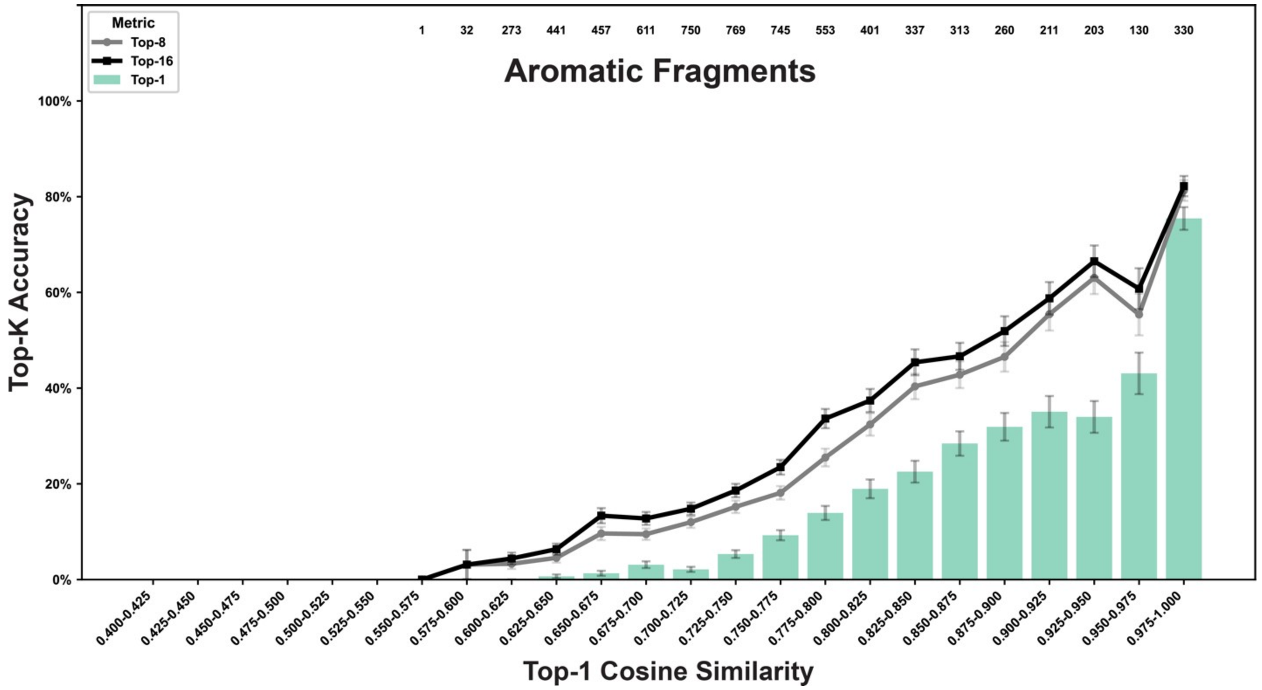

Figure S4. Relationship between Top-1 cosine similarity and Top-K accuracy for the aromatic-fragment model (≥ 4 heavy atoms). The plots show Top-1 (bars), Top-8 (gray line), and Top-16 (black line) accuracies binned by Top-1 cosine similarity. Numbers above each bin indicate the sample size. Error bars represent standard error.

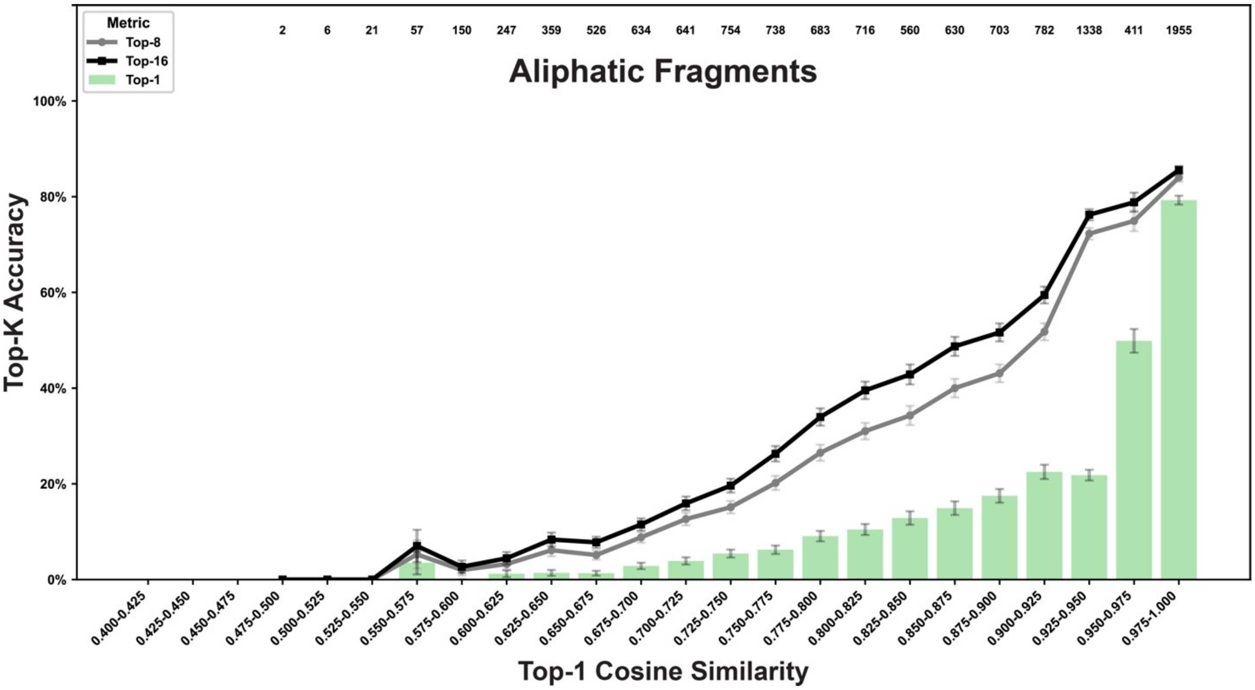

Figure S5. Relationship between Top-1 cosine similarity and Top-K accuracy for the aliphatic-fragment model (≥ 4 heavy atoms). The plots show Top-1 (bars), Top-8 (gray line), and Top-16 (black line) accuracies binned by Top-1 cosine similarity. Numbers above each bin indicate the sample size. Error bars represent standard error.

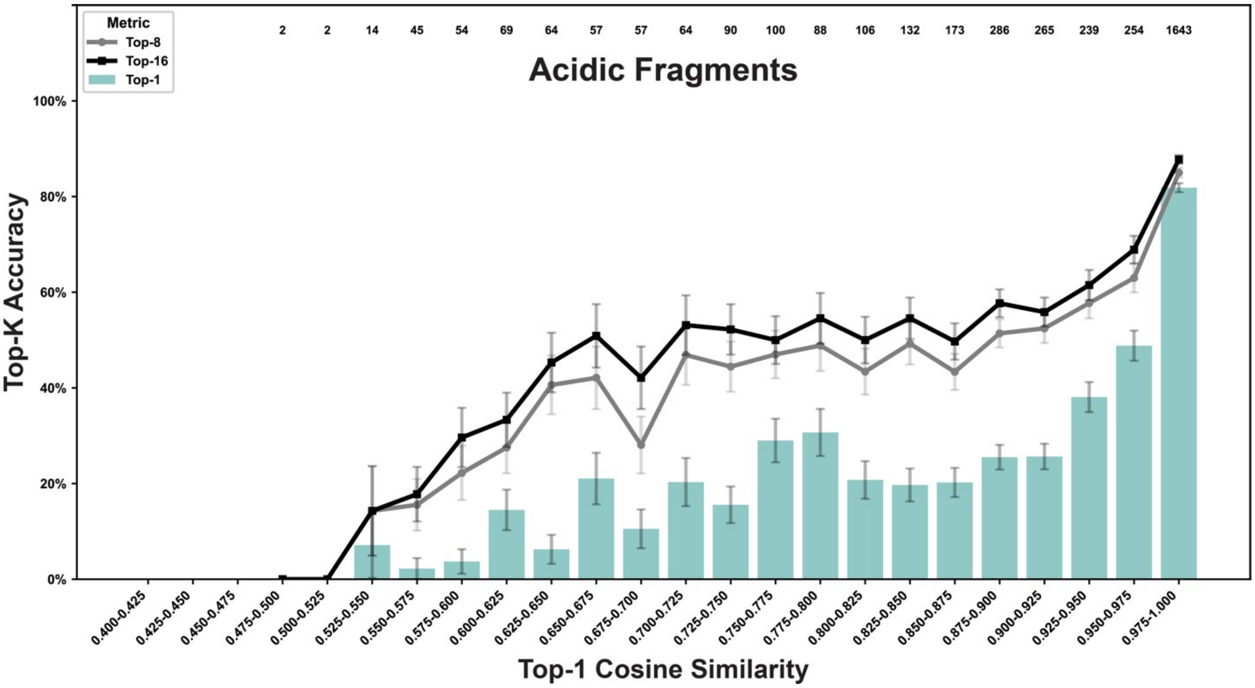

Figure S6. Relationship between Top-1 cosine similarity and Top-K accuracy for the acidic-fragment model (≥ 4 heavy atoms). The plots show Top-1 (bars), Top-8 (gray line), and Top-16 (black line) accuracies binned by Top-1 cosine similarity. Numbers above each bin indicate the sample size. Error bars represent standard error.

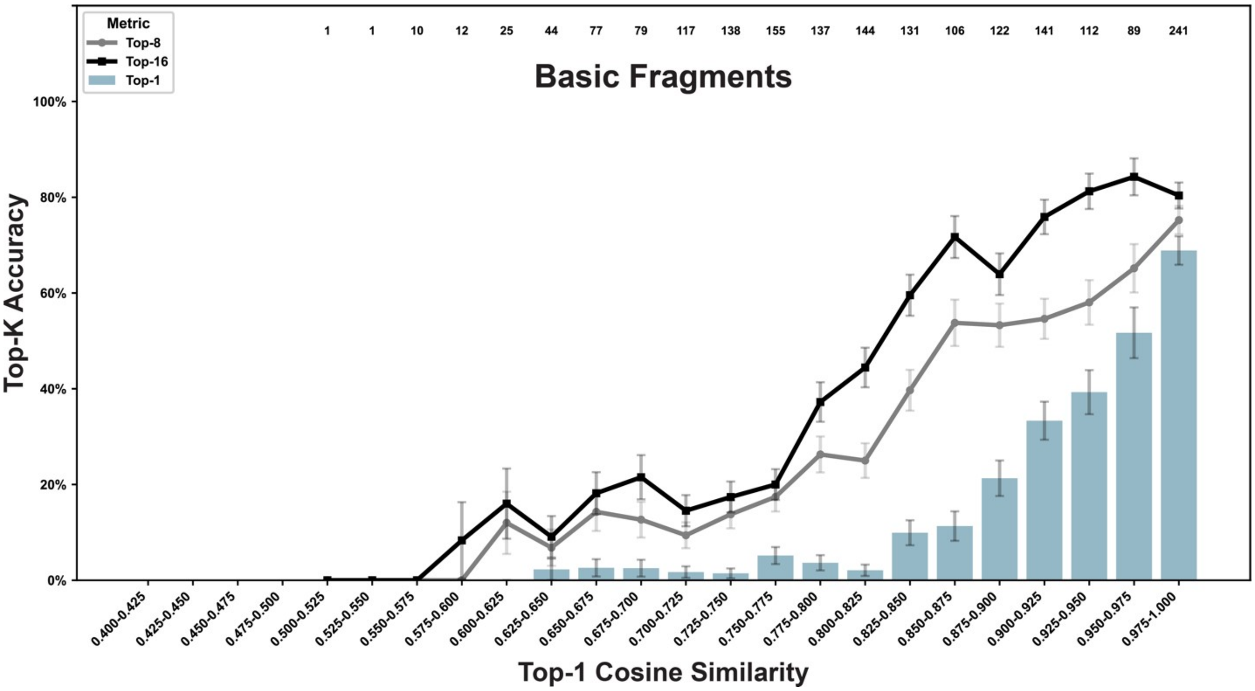

Figure S7. Relationship between Top-1 cosine similarity and Top-K accuracy for the basic-fragment model (≥ 4 heavy atoms). The plots show Top-1 (bars), Top-8 (gray line), and Top-16 (black line) accuracies binned by Top-1 cosine similarity. Numbers above each bin indicate the sample size. Error bars represent standard error.

Accuracy of fragment-specific predictions as a function of cosine similarity

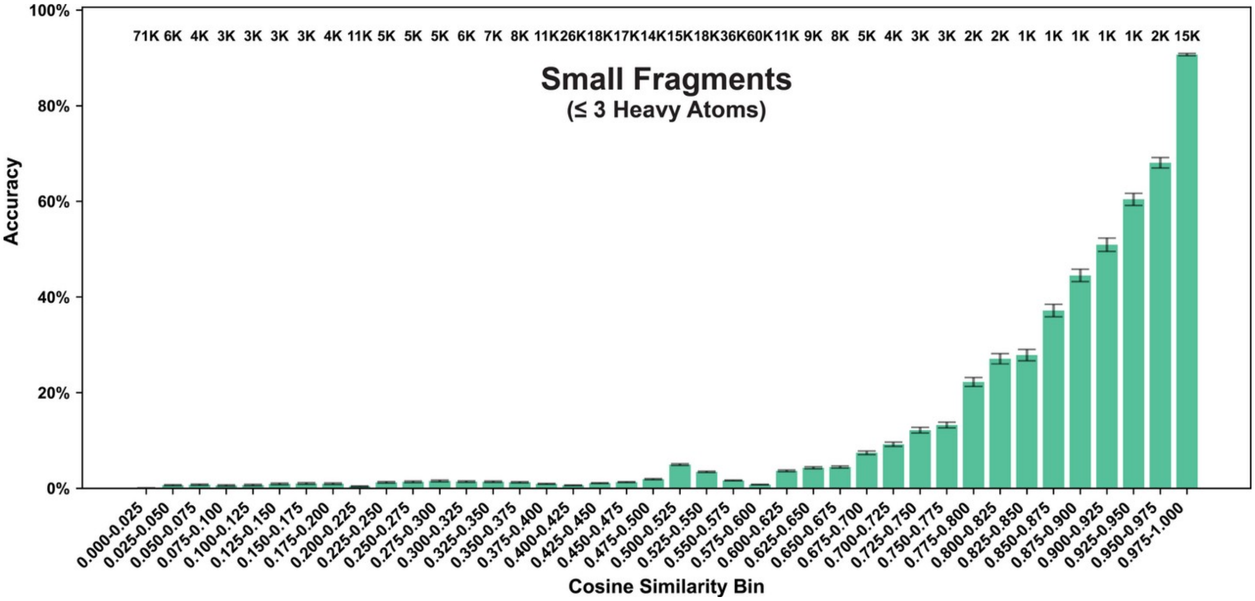

Figure S8. Relationship between a fragment's cosine-similarity score and its likelihood of matching the corresponding ground-truth fragment, when using the small-fragment model (≤ 3 heavy atoms) and a label set comprised of all small fragments from the testing set. Source data includes all Top-16 fragment selections from all small-fragment testing-set examples. The plots show accuracy (bars) binned by cosine similarity between DeepFrag-predicted and label-set fingerprints, where accuracy represents the percentage of cases in which the recommended fragment matched the ground-truth fragment within each bin. Numbers above each bin indicate the sample size, abbreviated where necessary (K represents thousands). Error bars represent standard error.

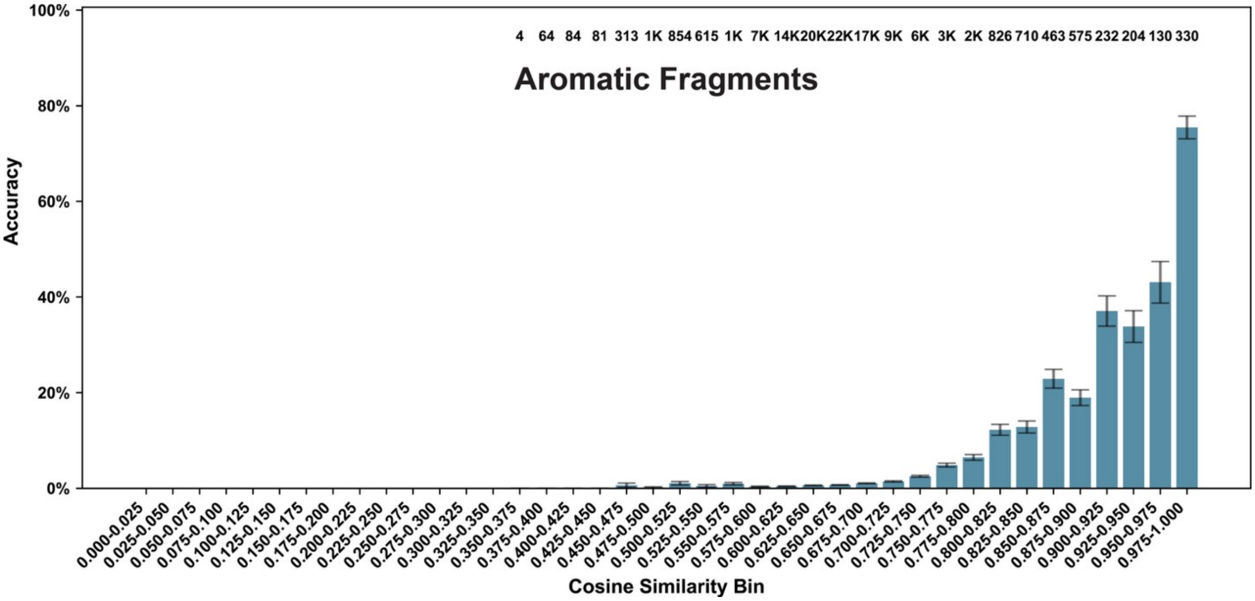

Figure S9. Relationship between a fragment's cosine-similarity score and its likelihood of matching the corresponding ground-truth fragment, when using the aromatic-fragment model (≥ 4 heavy atoms) and a label set comprised of all aromatic fragments from the testing set. Source data includes all Top-16 fragment selections from all aromatic-fragment testing-set examples. The plots show accuracy (bars) binned by cosine similarity between DeepFrag-predicted and label-set fingerprints, where accuracy represents the percentage of cases in which the recommended fragment matched the ground-truth fragment within each bin. Numbers above each bin indicate the sample size, abbreviated where necessary (K represents thousands). Error bars represent standard error.

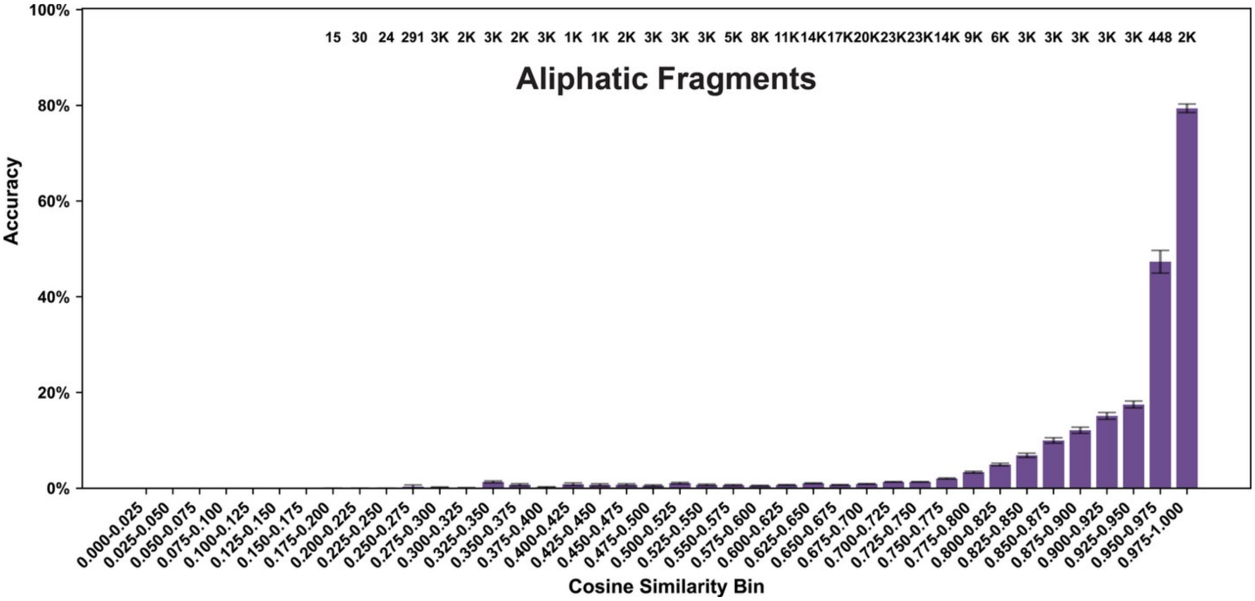

Figure S10. Relationship between a fragment's cosine-similarity score and its likelihood of matching the corresponding ground-truth fragment, when using the aliphatic-fragment model (≥ 4 heavy atoms) and a label set comprised of all aliphatic fragments from the testing set. Source data includes all Top-16 fragment selections from all aliphatic-fragment testing-set examples. The plots show accuracy (bars) binned by cosine similarity between DeepFrag-predicted and label-set fingerprints, where accuracy represents the percentage of cases in which the recommended fragment matched the ground-truth fragment within each bin. Numbers above each bin indicate the sample size, abbreviated where necessary (K represents thousands). Error bars represent standard error.

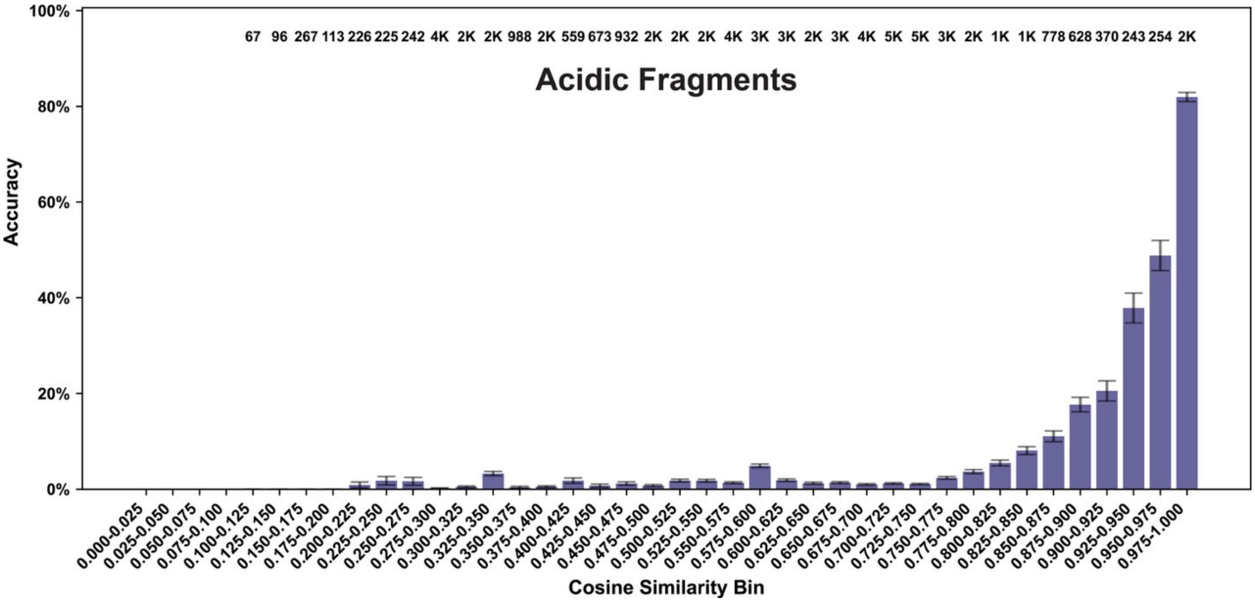

Figure S11. Relationship between a fragment's cosine-similarity score and its likelihood of matching the corresponding ground-truth fragment, when using the acidic-fragment model (≥ 4 heavy atoms) and a label set comprised of all acidic fragments from the testing set. Source data includes all Top-16 fragment selections from all acidic-fragment testing-set examples. The plots show accuracy (bars) binned by cosine similarity between DeepFrag-predicted and label-set fingerprints, where accuracy represents the percentage of cases in which the recommended fragment matched the ground-truth fragment within each bin. Numbers above each bin indicate the sample size, abbreviated where necessary (K represents thousands). Error bars represent standard error.

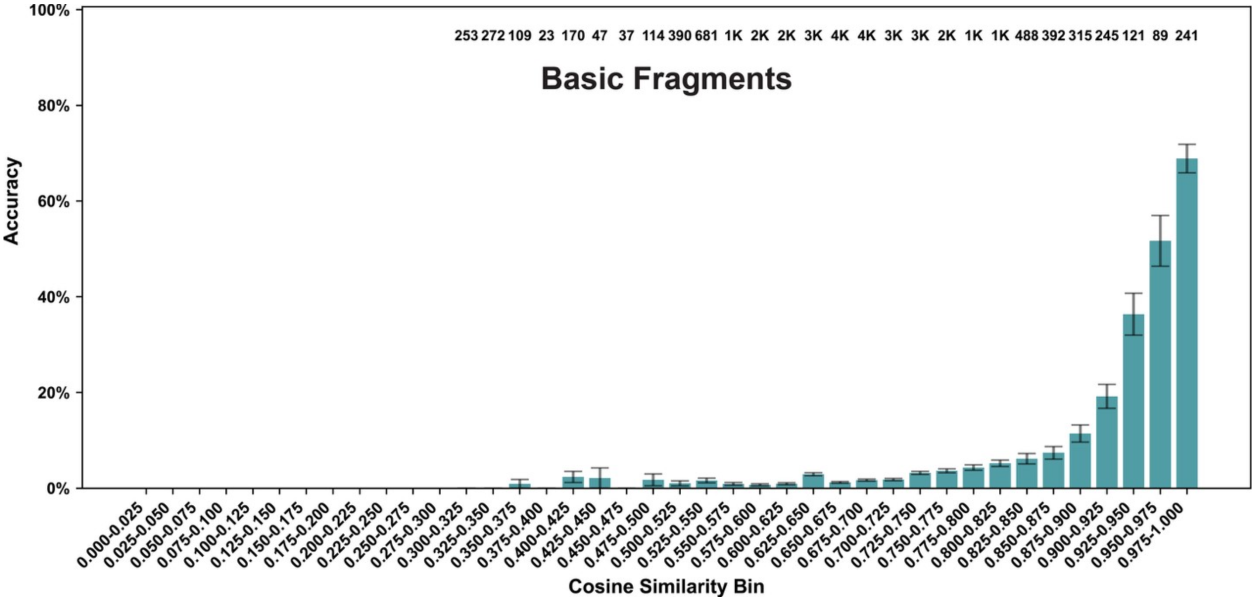

Figure S12. Relationship between a fragment's cosine-similarity score and its likelihood of matching the corresponding ground-truth fragment, when using the basic-fragment model (≥ 4 heavy atoms) and a label set comprised of all basic fragments from the testing set. Source data includes all Top-16 fragment selections from all basic-fragment testing-set examples. The plots show accuracy (bars) binned by cosine similarity between DeepFrag-predicted and label-set fingerprints, where accuracy represents the percentage of cases in which the recommended fragment matched the ground-truth fragment within each bin. Numbers above each bin indicate the sample size, abbreviated where necessary (K represents thousands). Error bars represent standard error.

Protein-specific finetuned DeepFrag models

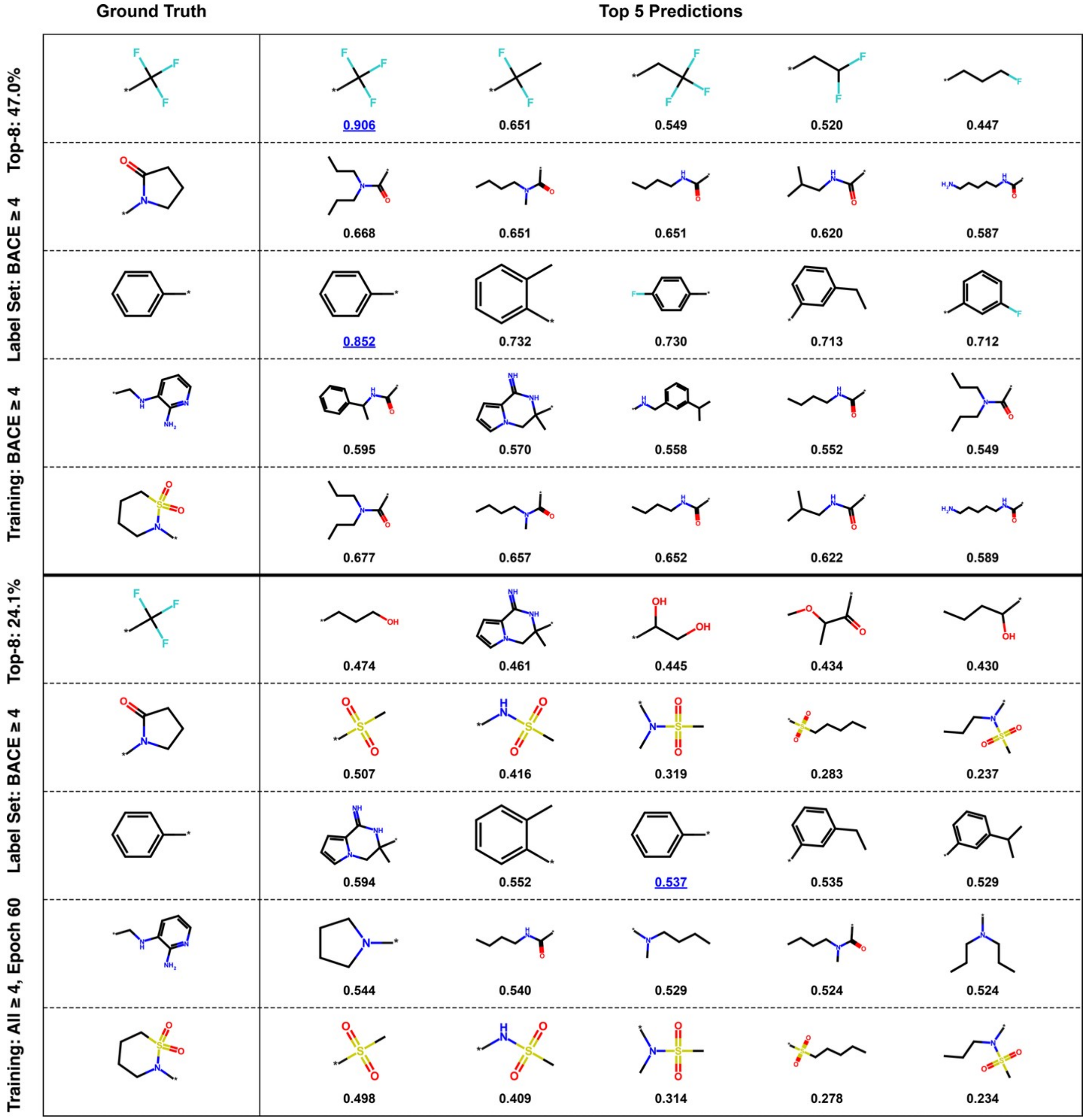

Figure S13. BACE fine-tuning. The upper five rows show randomly selected testing-set predictions after 30 generations of fine-tuning on beta-secretase 1 (BACE) protein/ligand complexes, with corresponding ground-truth fragments in the left most column. The fine-tuned model was trained to predict large fragments (≥ 4 heavy atoms). The bottom five rows show the predictions of the model before finetuning when applied to the same examples. This foundational model was trained on large fragments (independent of chemical property or protein target). In both cases, the label set includes only large fragments, derived from the BACE testing-set examples.

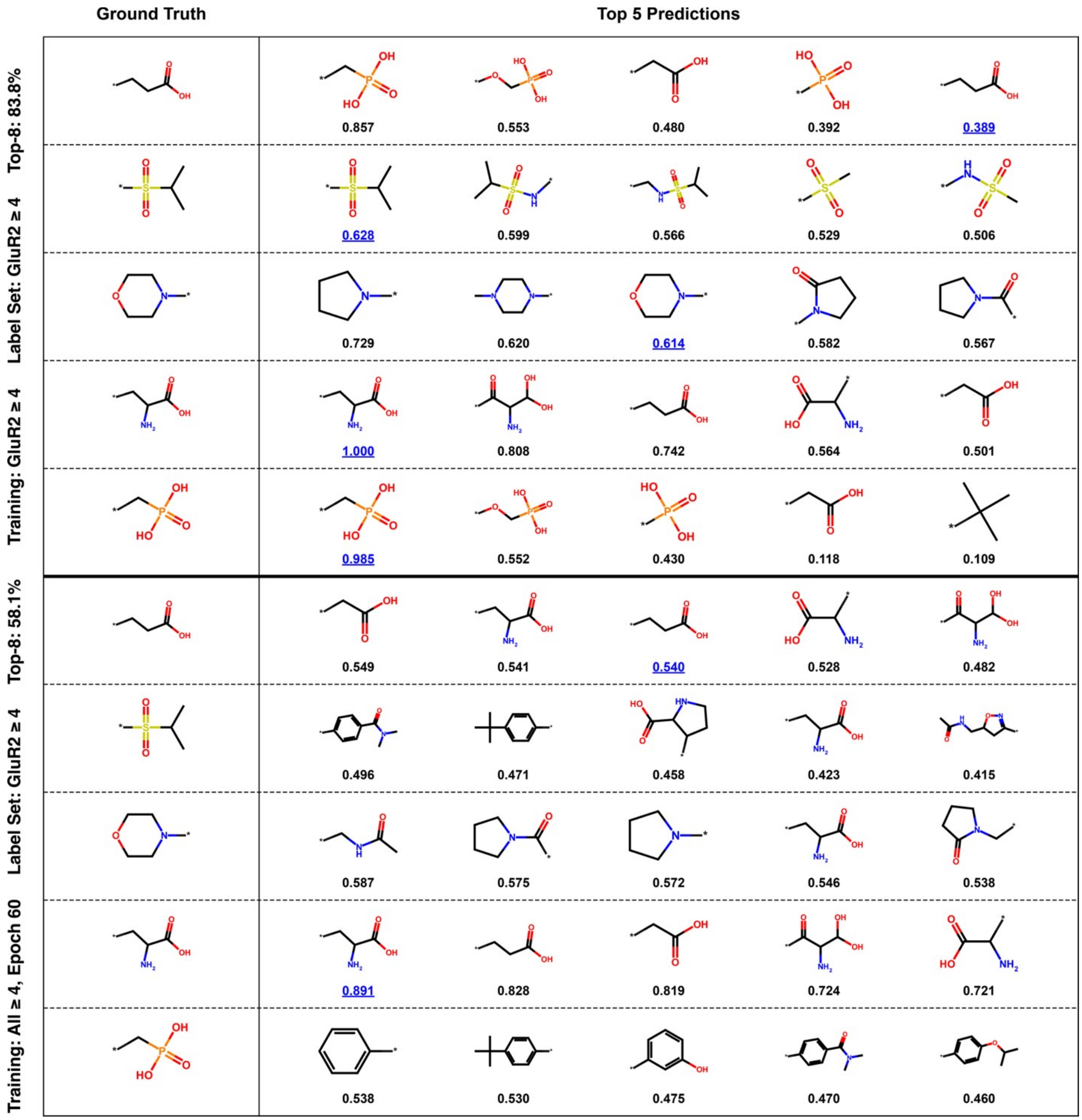

Figure S14. GluR2 fine-tuning. The upper five rows show randomly selected testing-set predictions after 30 generations of fine-tuning on glutamate receptor (GluR2) protein/ligand complexes, with corresponding ground-truth fragments in the left most column. The fine-tuned model was trained to predict large fragments (≥ 4 heavy atoms). The bottom five rows show the predictions of the model before finetuning when applied to the same examples. This foundational model was trained on large fragments (independent of chemical property or protein target). In both cases, the label set includes only large fragments, derived from the GluR2 testing-set examples.

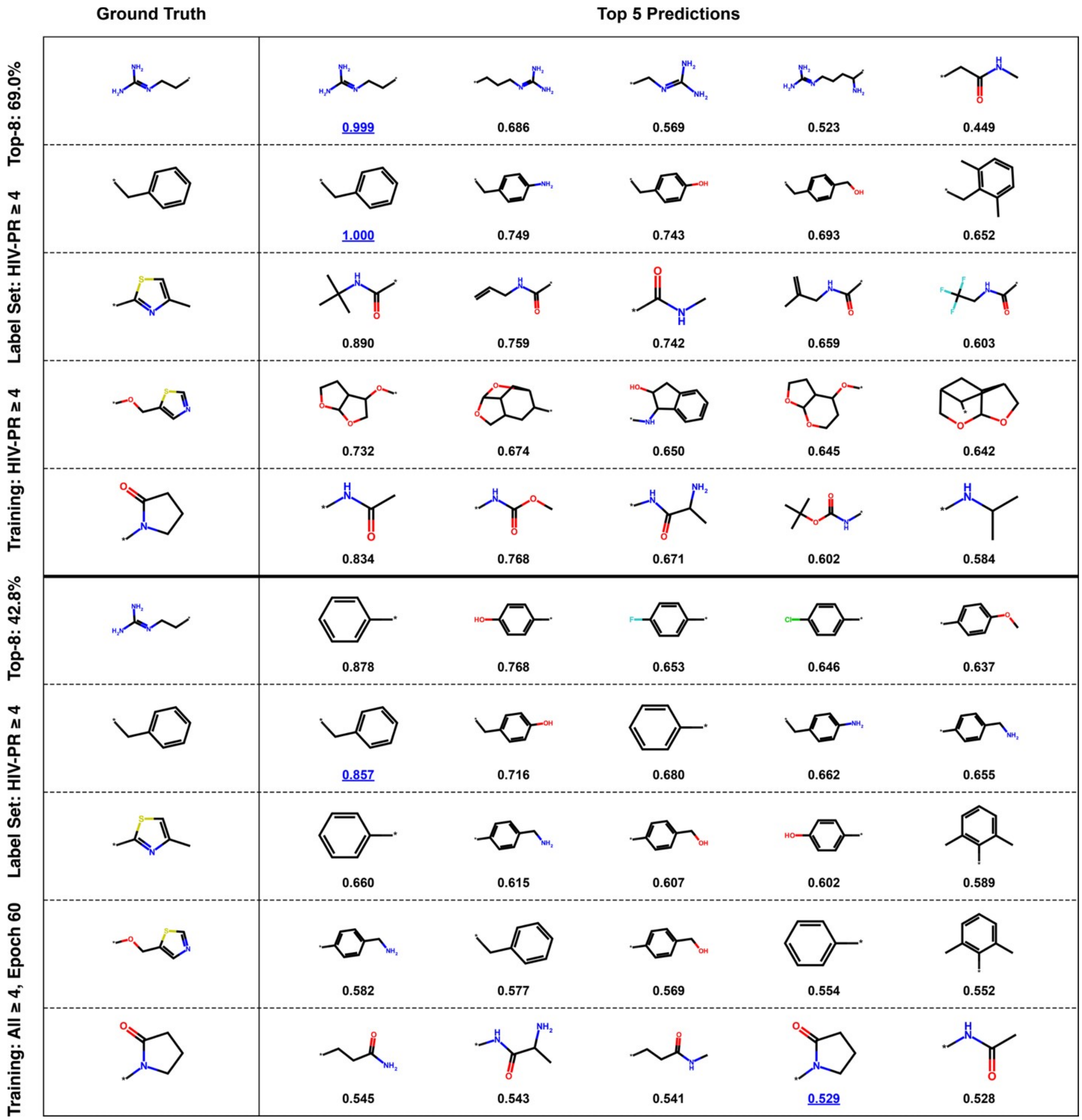

Figure S15. HIV-PR fine-tuning. The upper five rows show randomly selected testing-set predictions after 30 generations of fine-tuning on HIV-1 protease (HIV-PR) protein/ligand complexes, with corresponding ground-truth fragments in the left most column. The fine-tuned model was trained to predict large fragments (≥ 4 heavy atoms). The bottom five rows show the predictions of the model before finetuning when applied to the same examples. This foundational model was trained on large fragments (independent of chemical property or protein target). In both cases, the label set includes only large fragments, derived from the HIV-PR testing-set examples.

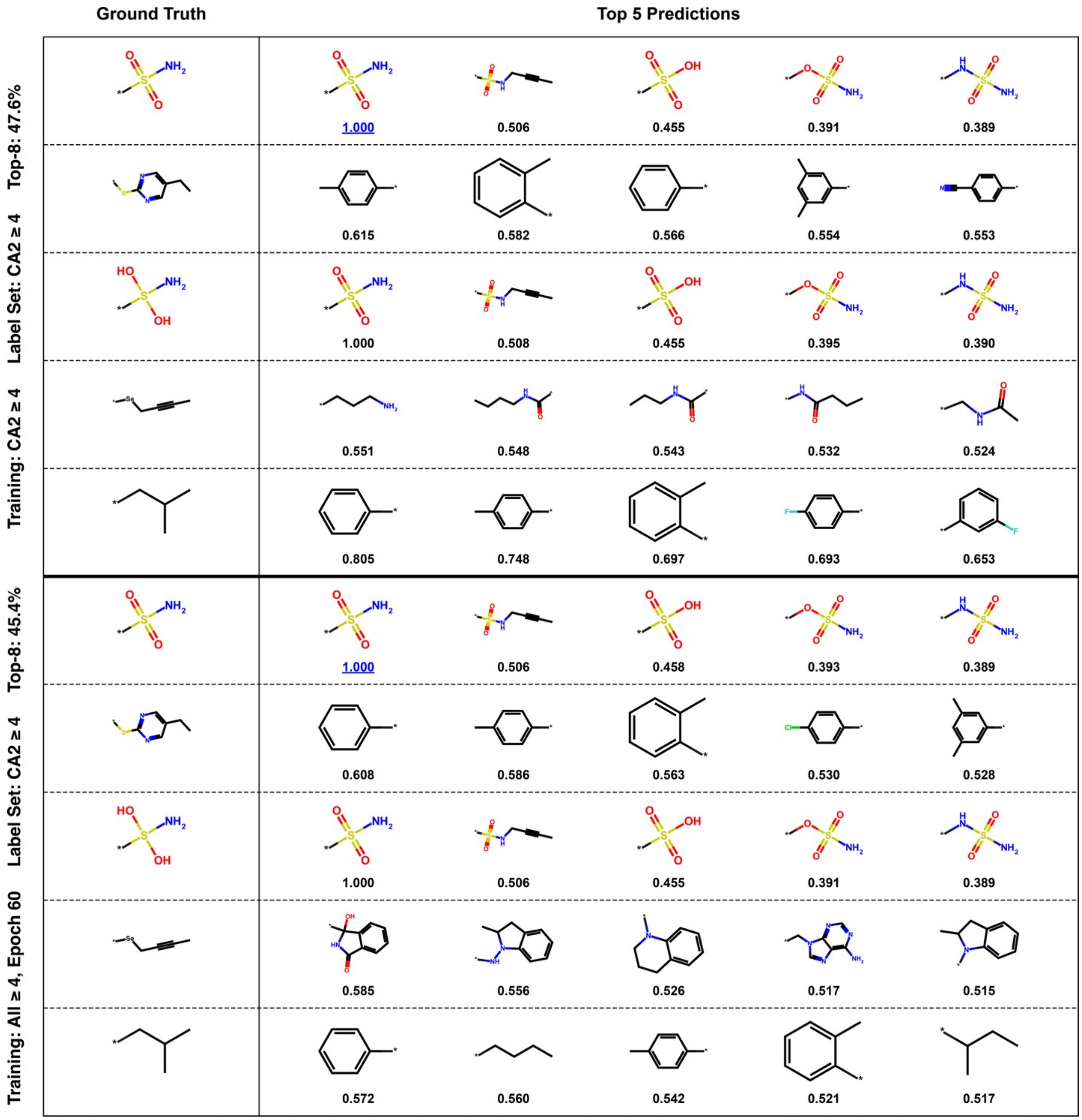

Figure S16. CA2 fine-tuning. The upper five rows show randomly selected testing-set predictions after 30 generations of fine-tuning on carbonic anhydrase 2 (CA2) protein/ligand complexes, with corresponding ground-truth fragments in the left most column. The fine-tuned model was trained to predict large fragments (≥ 4 heavy atoms). The bottom five rows show the predictions of the model before finetuning when applied to the same examples. This foundational model was trained on large fragments (independent of chemical property or protein target). In both cases, the label set includes only large fragments, derived from the CA2 testing-set examples.

References

(1) Russell, R. J.; Haire, L. F.; Stevens, D. J.; Collins, P. J.; Lin, Y. P.; Blackburn, G. M.; Hay, A. J.; Gamblin, S. J.; Skehel, J. J. The structure of H5N1 avian influenza neuraminidase suggests new opportunities for drug design. *Nature* **2006**, *443* (7107), 45-49. DOI: 10.1038/nature05114 From NLM Medline.

(2) Cheng, T. J.; Weinheimer, S.; Tarbet, E. B.; Jan, J. T.; Cheng, Y. S.; Shie, J. J.; Chen, C. L.; Chen, C. A.; Hsieh, W. C.; Huang, P. W.; et al. Development of oseltamivir phosphonate congeners as anti-influenza agents. *J Med Chem* **2012**, *55* (20), 8657-8670. DOI: 10.1021/jm3008486 From NLM Medline.

(3) Rogers, D.; Hahn, M. Extended-connectivity fingerprints. *J Chem Inf Model* **2010**, *50* (5), 742-754. DOI: 10.1021/ci100050t From NLM Medline.

(4) Durant, J. L.; Leland, B. A.; Henry, D. R.; Nourse, J. G. Reoptimization of MDL keys for use in drug discovery. *J Chem Inf Comput Sci* **2002**, *42* (6), 1273-1280. DOI: 10.1021/ci010132r From NLM Medline.
